# Bivariate power Lomax distribution with medical applications

**DOI:** 10.1371/journal.pone.0282581

**Published:** 2023-03-08

**Authors:** Maha E. Qura, Aisha Fayomi, Mutua Kilai, Ehab M. Almetwally

**Affiliations:** 1 Department of Statistics, Mathematics, and Insurance, Benha University, Benha, Egypt; 2 Department of Statistics, Faculty of Science, King Abdulaziz University, Jeddah, Saudi Arabia; 3 Pan African Institute of Basic Science, Technology and Innovation, Nairobi, Kenya; 4 Department of Statistical, Faculty of Business Administration, Delta University for Science and Technology, Gamasa, Egypt; University of Mauritius, MAURITIUS

## Abstract

In this paper, a bivariate power Lomax distribution based on Farlie-Gumbel-Morgenstern (FGM) copulas and univariate power Lomax distribution is proposed, which is referred to as BFGMPLx. It is a significant lifetime distribution for modeling bivariate lifetime data. The statistical properties of the proposed distribution, such as conditional distributions, conditional expectations, marginal distributions, moment-generating functions, product moments, positive quadrant dependence property, and Pearson’s correlation, have been studied. The reliability measures, such as the survival function, hazard rate function, mean residual life function, and vitality function, have also been discussed. The parameters of the model can be estimated through maximum likelihood and Bayesian estimation. Additionally, asymptotic confidence intervals and credible intervals of Bayesian’s highest posterior density are computed for the parameter model. Monte Carlo simulation analysis is used to estimate both the maximum likelihood and Bayesian estimators.

## 1 Introduction

The Lomax (Lx) [1], or Pareto II, distribution presented primarily for modeling business failure data. In statistical literature, many authors used this distribution to model reliability data set and life testing [2], income and wealth data sets [3], biological sciences [4], and data set from receiver operating characteristic (ROC) curves analysis [5].

Rady et al. [6] proposed power lomax (PLx) model as a new extension of the Lx distribution with an extra shape parameter and applied it to medical databases. The cumulative distribution function (CDF) and probability density function (PDF) of power lomax are

$$\begin{array}{c} F\left(x;\gamma ,\beta ,\lambda \right)=1-\lambda^{\gamma }{\left(\lambda +x^{\beta }\right)}^{-\gamma },\;\;\gamma ,\beta ,\lambda >0,\;\;x>0, \end{array}$$ (1)

and

$$\begin{array}{c} f\left(x;\gamma ,\beta ,\lambda \right)=\gamma \beta \lambda^{\gamma }x^{\beta -1}{\left(\lambda +x^{\beta }\right)}^{-\gamma -1},\;\;\gamma ,\beta ,\lambda >0,\;\;x>0, \end{array}$$ (2)

where *γ*, *β* is shape parameter and λ is scale parameter.

In statistical literature, there are various ways to construct bivariate distributions, and copulas are one of them (see Lai [7] and Nelsen [8]). Copulas are a useful tool for describing a bivariate distribution with dependence structure. They are defined by Nelsen [8] as a function that joins bivariate distribution functions with uniform [0, 1] margins. Sklar [9] presents the joint PDF and joint CDF for two marginal univariate distributions as follows: If *F*(*x*_*i*_) is the univariate CDF of *X*_*i*_, *i* = 1, 2, the joint CDF and PDF, denoted by *F*(*x*_1_, *x*_2_) and *f*(*x*_1_, *x*_2_), are defined by the copula function, given as

$$\begin{array}{c} F\left(x_{1},x_{2}\right)=C_{\theta }\left(F_{1}\left(x_{1}\right),F_{2}\left(x_{2}\right)\right), \end{array}$$ (3)

where *θ* is the dependence measures between *X*_1_ and *X*_2_, *C* is copula’s cdf, and *c* is copula’s pdf.

$$\begin{array}{c} f\left(x_{1},x_{2}\right)=f_{1}\left(x_{1}\right)f_{2}\left(x_{2}\right)C_{\theta }\left(f_{1}\left(x_{1}\right),f_{2}\left(x_{2}\right)\right). \end{array}$$ (4)

Bivariate distribution studies can be advanced through the use of copulas, which model the relationship between two random variables. Popular copulas include Gaussian, Clayton, Farlie-Gumbel-Morgenstern, Gumbel, Frank, and Archimedean copulas. Further details can be found in the references, [10–17]. The choice of copula function depends on the type of dependence structure between the two random variables. For example, the Gaussian copula is used to model linear dependence while the Clayton copula is used to model positive dependence.

The Farlie-Gumbel-Morgenstern (FGM) have been characterized using Eqs (3) and (4). Morgenstern [18] proposed a simple method for constructing a bivariate family of distributions using marginals. Farlie [19] proposed a generalization of Morgenstern’s method that is known as the Farlie-Gumbel-Morgenstern (FGM) family of distributions. The FGM copula offers several advantages in modeling bivariate distributions. One of the main advantages is its flexibility in capturing a wide range of dependence structures, from complete independence to perfect dependence [14]. Additionally, the FGM copula is capable of handling asymmetrical dependence, making it well-suited for modeling data with skewed or heavy-tailed distributions [18]. Moreover, the FGM copula allows for the construction of bivariate distributions with a wide range of marginals, including continuous and discrete marginals [8]. Furthermore, the FGM copula has a simple form, which makes it computationally efficient and easy to implement in practice [10]. A new bivariate model based on adaptive progressive hybrid censored has been introduced by [11]. Bivariate Chen distribution based on FGM copula has been obtained by [12]. The bivariate models based on copula function with application of accelerated life testing (ALT) has been suggested by [13]. These features make the FGM copula a useful tool for many real-world applications, particularly in the fields of finance, insurance, and medical research [20].

A lot of work has been done in bivariate distributions based on Morgenstern-type distributions. Gupta et al. [21] derived three- and five-parameter bivariate beta distributions from the Morgenstern system of curves and studied the distributions of the product and quotient of variates. Vaidyanathan et al. [22] proposed a bivariate Lindley distribution using the Morgenstern method and presented some properties. Almetwally et al. [23] proposed bivariate distributions called the FGM Bivariate Fréchet (FGMBF) and AMH Bivariate Fréchet (AMHBF) distributions using Farlie-Gumbel-Morgenstern (FGM) and Ali-Mikhail-Haq (AMH) copulas and univariate Fréchet distributions. El-Sherpieny et al. [24] proposed the bivariate FGM Weibull-G family, which is a new flexible bivariate generalized family of distributions based on the FGM copula. Muhammed et al. [25] presented the bivariate inverted Topp-Leone (BITL) distribution, which is derived from Farlie-Gumbel-Morgenstern, Ali-Mikhail-Haq, Plackett, and Clayton copulas. Abulebda et al. [26] introduced a new bivariate XGamma (BXG) distribution and investigated its statistical properties through examination of real data. Hassan et al. [27] proposed the bivariate generalized half-logistic distribution using the FGM copula to asses household financial affordability in the Kingdom of Saudi Arabia.

Our motivation for conducting this research is to

1. propose a new bivariate called bivariate Farlie-Gumbel-Morgenstern power Lomax (BFGMPLx) distribution based on the Farlie-Gumbel-Morgenstern approach that overcomes limitations of existing distributions in modeling complex data sets.
2. Improve the modeling of complex data. The proposed distribution provides a more flexible and sophisticated method for modeling complex data sets, leading to improved predictions and decision-making.
3. Robustly handle heavy-tailed and skewed data. The new bivariate power Lomax distribution offers improved robustness and accuracy in handling such data, making it an ideal tool for various real-world applications.
4. gain new insights into the relationship between variables in medical data and the potential for improved patient outcomes.

The novelty of this research lies in the combination of the power Lomax distribution and the FGM copula. This new distribution addresses the limitations of prior work by offering improved robustness and accuracy in modeling heavy-tailed or skewed data. Additionally, the use of the FGM copula provides more flexibility in modeling of a variety of different types of dependence structures, including both positive and negative dependence between the two variables compared to traditional copula functions. This makes it a good choice for modeling data with complex relationships

The paper is structured as follows: In section 2, the description and notation of BFGMPLx distribution is introduced. Section 3 discusses the statistical properties of BFGMPLx. In section 4, we study the positive (negative) quadrant dependence property of BFGMPLx. In section 5, different reliability measures for BFGMPLx are obtained. The maximum likelihood (ML) and Bayesian methods are used to estimate the parameters in BFGMPLx in section 6. Section 7 covers asymptotic and credible intervals. Simulation and an application to real data are provided in sections 8 and 9, respectively. Finally, the paper is concluded in section 10.

## 2 Bivariate Farlie-Gumble-Morgenstern power Lomax distribution

The joint CDF and PDF of the FGM copula [28] are given, respectively, by

$$\begin{array}{ccc} F(x_{1},x_{2}) & = & F_{1}\left(x_{1}\right)F_{2}\left(x_{2}\right)[1+\theta (1-F_{1}\left(x_{1}\right))(1-F_{2}\left(x_{2}\right))], \end{array}$$ (5)

$$\begin{array}{ccc} f(x_{1},x_{2}) & = & f_{1}\left(x_{1}\right)f_{2}\left(x_{2}\right)[1+\theta (1-2F_{1}\left(x_{1}\right))(1-2F_{2}\left(x_{2}\right))], \end{array}$$ (6)

where −1 < *θ* < 1. The random variables say (*X*_1_, *X*_2_) follow the BFGMPLx distribution if its CDF is defined by

$$\begin{array}{c} \begin{array}{cccc} F(x_{1},x_{2})= & \left[1-\lambda_{1}^{\gamma_{1}}{\left(\lambda_{1}+x_{1}^{\beta_{1}}\right)}^{-\gamma_{1}}][1-\lambda_{2}^{\gamma_{2}}{\left(\lambda_{2}+x_{2}^{\beta_{2}}\right)}^{-\gamma_{2}}\right] & & \\ & [1+\theta \lambda_{1}^{\gamma_{1}}{\left(\lambda_{1}+x_{1}^{\beta_{1}}\right)}^{-\gamma_{1}}\lambda_{2}^{\gamma_{2}}{\left(\lambda_{2}+x_{2}^{\beta_{2}}\right)}^{-\gamma_{2}}], \end{array} \end{array}$$ (7)

where λ_1_, *β*_1_, *γ*_1_, λ_2_, *β*_2_, *γ*_2_ > 0, −1 < *θ* < 1, and *x*_1_, *x*_2_ > 0. The corresponding PDF is

$$\begin{array}{c} \begin{array}{cccc} f(x_{1},x_{2})= & \left[\gamma_{1}\beta_{1}\lambda_{1}^{\gamma_{1}}x_{1}^{\beta_{1}-1}{\left(\lambda_{1}+x_{1}^{\beta_{1}}\right)}^{-\gamma_{1}-1}][\gamma_{2}\beta_{2}\lambda_{2}^{\gamma_{2}}x_{2}^{\beta_{2}-1}{\left(\lambda_{2}+x_{2}^{\beta_{2}}\right)}^{-\gamma_{2}-1}\right] & & \\ & [1+\theta (2\lambda_{1}^{\gamma_{1}}{\left(\lambda_{1}+x_{1}^{\beta_{1}}\right)}^{-\gamma_{1}}-1)(2\lambda_{2}^{\gamma_{2}}{\left(\lambda_{2}+x_{2}^{\beta_{2}}\right)}^{-\gamma_{2}}-1)]. \end{array} \end{array}$$ (8)

**Fig 1:**
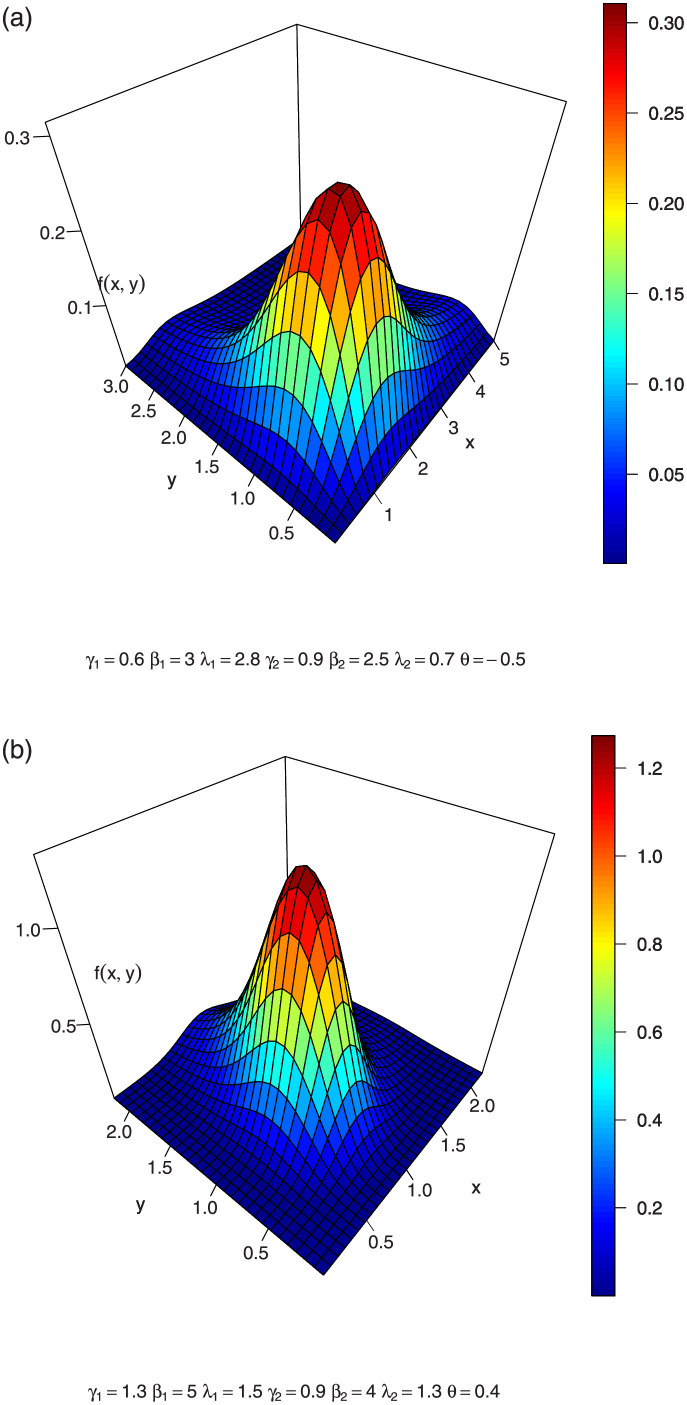
3-dimension of joint density of BFGMPLx.

**Fig 2:**
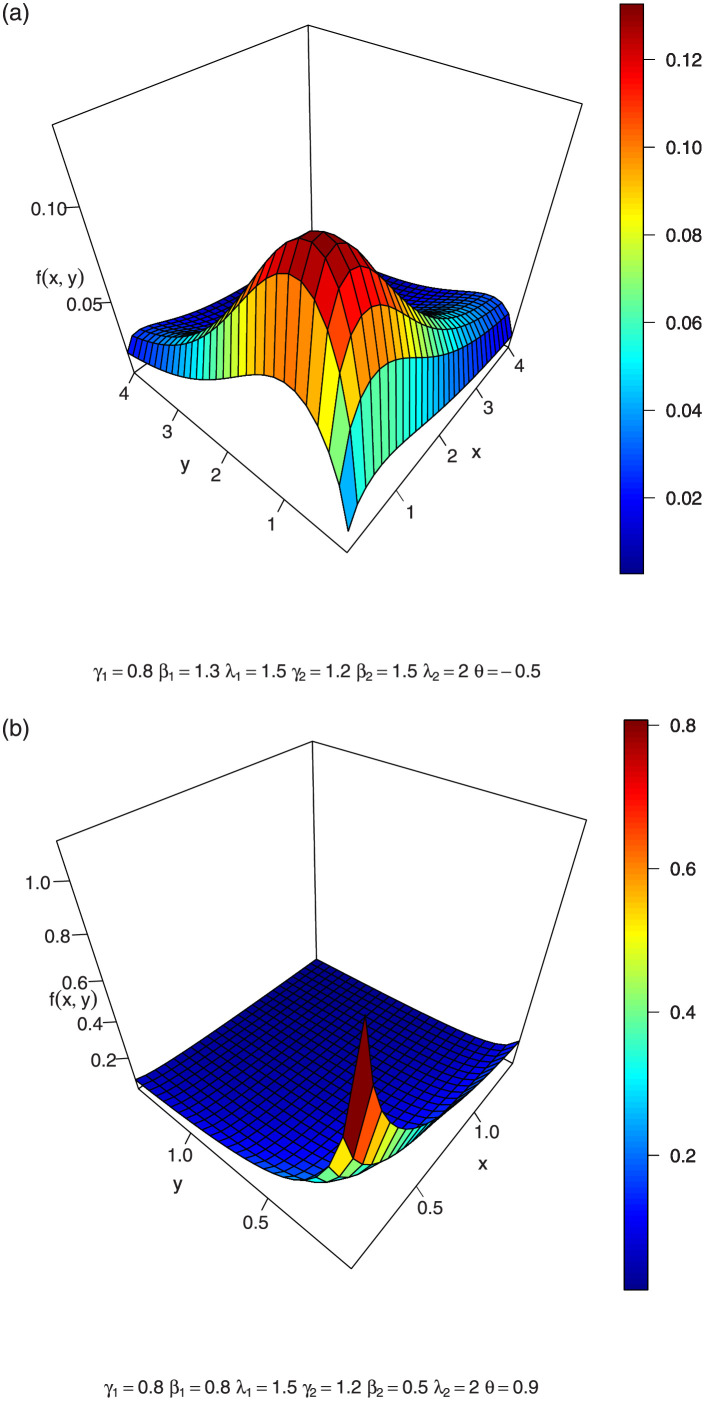
3-dimension of joint density of BFGMPLx.

**Fig 3:**
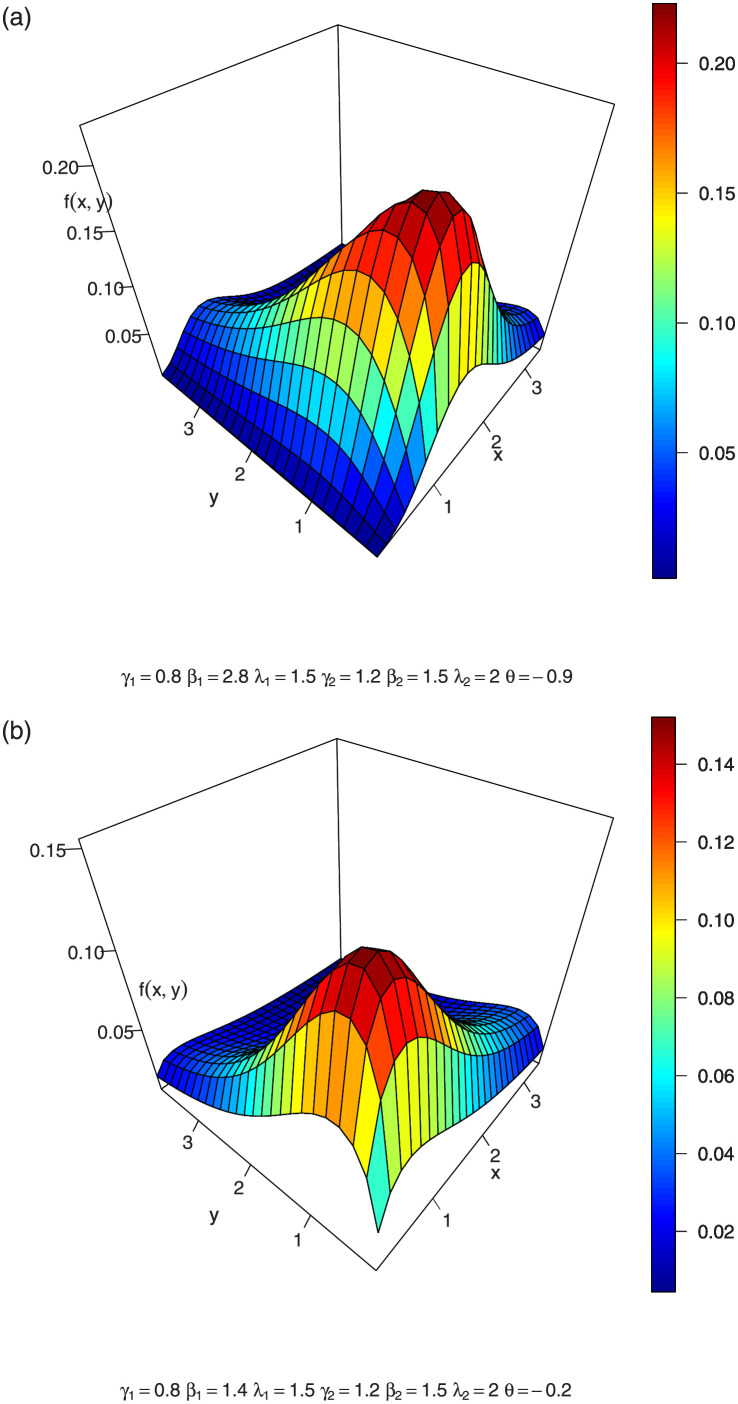
3-dimension of joint density of BFGMPLx.

In Figs 1–3, we show the 3-dimension plots of the joint density of a BFGMPLx distribution for various parameter values.

Sreelakshmi [29] introduced the relationship between copula and reliability copula which is defined as follows:

$$\begin{array}{c} R\left(x_{1},x_{2}\right)=1-F_{1}\left(x_{1}\right)-F_{2}\left(x_{2}\right)+C\left(F_{1}\left(x_{1}\right),F_{2}\left(x_{2}\right)\right). \end{array}$$ (9)

According to (9), The FGM reliability is

$$\begin{array}{c} R\left(x_{1},x_{2}\right)=1-F_{1}\left(x_{1}\right)-F_{2}\left(x_{2}\right)+F_{1}\left(x_{1}\right)F_{2}\left(x_{2}\right)[1+\theta (1-F_{1}\left(x_{1}\right))(1-F_{2}\left(x_{2}\right))]. \end{array}$$

The following is the reliability function for BFGMPLx distribution:

$$\begin{array}{c} \begin{array}{cccc} R(x_{1},x_{2})= & \left[\lambda_{1}^{\gamma_{1}}{\left(\lambda_{1}+x_{1}^{\beta_{1}}\right)}^{-\gamma_{1}}][\lambda_{2}^{\gamma_{2}}{\left(\lambda_{2}+x_{2}^{\beta_{2}}\right)}^{-\gamma_{2}}\right] & & \\ & [1+\theta (1-\lambda_{1}^{\gamma_{1}}{\left(\lambda_{1}+x_{1}^{\beta_{1}}\right)}^{-\gamma_{1}})(1-\lambda_{2}^{\gamma_{2}}{\left(\lambda_{2}+x_{2}^{\beta_{2}}\right)}^{-\gamma_{2}})]. \end{array} \end{array}$$ (10)

## 3 Properties of BFGMPLx distribution

In this section, some important statistical properties of the BFGMPLx distribution are introduced such as marginal distributions, conditional distributions, conditional expectations, product moments and moment generating function.

### 3.1 The marginal distributions

The functions of marginal density for *X*_1_ and *X*_2_, respectively,

$$\begin{array}{c} f\left(x_{1};\gamma_{1},\beta_{1},\lambda_{1}\right)=\gamma_{1}\beta_{1}\lambda_{1}^{\gamma_{1}}x_{1}^{\beta_{1}-1}{\left(\lambda_{1}+x_{1}^{\beta_{1}}\right)}^{-\gamma_{1}-1},\;\;\gamma_{1},\beta_{1},\lambda_{1}>0,\;\;x_{1}>0. \end{array}$$ (11)

$$\begin{array}{c} f\left(x_{2};\gamma_{2},\beta_{2},\lambda_{2}\right)=\gamma_{2}\beta_{2}\lambda_{2}^{\gamma_{2}}x_{2}^{\beta_{2}-1}{\left(\lambda_{2}+x_{2}^{\beta_{2}}\right)}^{-\gamma_{2}-1},\;\;\gamma_{2},\beta_{2},\lambda_{2}>0,\;\;x_{2}>0, \end{array}$$ (12)

which are Power Lomax distributed as shown in Eqs (11) and (12).

### 3.2 Conditional distribution

The distribution of conditional probability for *X*_2_ provided *X*_1_ is obtained as follows

$$\begin{array}{c} \begin{array}{cccc} f(x_{2}|x_{1})= & \gamma_{2}\beta_{2}\lambda_{2}^{\gamma_{2}}x_{2}^{\beta_{2}-1}{\left(\lambda_{2}+x_{2}^{\beta_{2}}\right)}^{-\gamma_{2}-1} & & \\ & [1+\theta -2\theta F_{2}\left(x_{2}\right)-2\theta F_{1}\left(x_{1}\right)(1-2F_{2}\left(x_{2}\right))]. \end{array} \end{array}$$ (13)

The conditional CDF of *X*_2_ given *X*_1_ is as follows:

$$\begin{array}{c} F\left(x_{2}|x_{1}\right)=F_{2}\left(x_{2}\right)[1+\theta -\theta F_{2}\left(x_{2}\right)-2\theta F_{1}\left(x_{1}\right)(1-F_{2}\left(x_{2}\right))]. \end{array}$$ (14)

The conditional probability distribution of *X*_1_ given *X*_2_ is derived as follows:

$$\begin{array}{c} \begin{array}{cccc} f(x_{1}|x_{2}) & =\gamma_{1}\beta_{1}\lambda_{1}^{\gamma_{1}}x_{1}^{\beta_{1}-1}{\left(\lambda_{1}+x_{1}^{\beta_{1}}\right)}^{-\gamma_{1}-1} & & \\ & [1+\theta -2\theta F_{2}\left(x_{2}\right)-2\theta F_{1}\left(x_{1}\right)(1-2F_{2}\left(x_{2}\right))]. \end{array} \end{array}$$ (15)

The conditional CDF of *X*_1_ given *X*_2_ is as follows:

$$\begin{array}{c} F\left(x_{1}|x_{2}\right)=F_{1}\left(x_{1}\right)[1+\theta -\theta F_{1}\left(x_{1}\right)-2\theta F_{2}\left(x_{2}\right)(1-F_{1}\left(x_{1}\right))]. \end{array}$$ (16)

The conditional expectation of *X*_1_ given *X*_2_ = *x*_2_ in BFGMPLx using the conditional density of *X*_1_ given *X*_2_ = *x*_2_ in (15) is calculated as

$$\begin{array}{c} \begin{array}{cccc} E(x_{1}|x_{2}) & =\frac{\lambda_{1}^{\frac{1}{\beta_{1}}}}{\beta_{1}}B(\frac{1}{\beta_{1}},\gamma_{1}-\frac{1}{\beta_{1}}) & & \\ & [1+\theta -2\theta F_{2}\left(x_{2}\right)+(1-\frac{\Lambda_{1}}{2})(4\theta F_{2}\left(x_{2}\right)-2\theta )], \end{array} \end{array}$$ (17)

where $\Lambda_{j}=\frac{B(\frac{1}{\beta_{j}},2\gamma_{j}-\frac{1}{\beta_{j}})}{B(\frac{1}{\beta_{j}},\gamma_{j}-\frac{1}{\beta_{j}})}$, *j* = 1, 2 and B(a,b) is beta function with *a* and *b* which are two real numbers greater than 0.

The above conditional expectation is non-linear in *X*_2_, as can be seen. In a similar manner it can be demonstrated that the conditional expectation of *X*_2_ given *X*_1_ = *x*_1_ is also non-linear in *x*_1_.

### 3.3 Generating random variables

By (14), a bivariate sample of the Power Lomax distribution based on the conditional method can be generated:

1. Generate U and V independently from a Uniform(0, 1) distribution.
2. Set $x_{1}=Q_{BFGMPLx}=G^{-1}[\lambda_{1}({\left(1-U\right)}^{-\frac{1}{\gamma_{1}}}-1){]}^{\frac{1}{\beta_{1}}}$.
3. *F*(*x*_2_ ∣ *x*_1_) = *V* in (14) to obtain *x*_2_ using numerical analysis such as Newton Raphson, and etc.
4. Repeat 1-3 (n) times to get (*x*_1*i*_, *x*_2*i*_), *i* = 1, 2, …., *n*.

### 3.4 Moment generating function

Let (*X*_1_, *X*_2_) represent a random variable that its PDF defined in Eq (8). The moment generating function of (*X*_1_, *X*_2_) is then obtained by

$$\begin{array}{c} \begin{array}{cccc} M_{x_{1},x_{2}}\left(t_{1},t_{2}\right)= & \sum_{n_{1}=0}^{\infty }\frac{{\left(t_{1}\right)}^{n_{1}}}{n_{1}!}\frac{n_{1}}{\beta_{1}}\lambda_{1}^{\frac{n_{1}}{\beta_{1}}}B\left(\frac{n_{1}}{\beta_{1}},\gamma_{1}-\frac{n_{1}}{\beta_{1}}\right)\sum_{n_{2}=0}^{\infty }\frac{{\left(t_{2}\right)}^{n_{2}}}{n_{2}!}\frac{n_{2}}{\beta_{2}}\lambda_{2}^{\frac{n_{2}}{\beta_{2}}} & & \\ & B\left(\frac{n_{2}}{\beta_{2}},\gamma_{2}-\frac{n_{2}}{\beta_{2}}\right)[1+\theta -2\theta \Omega_{2}-2\theta \Omega_{1}+4\theta \Omega_{1}\Omega_{2}], \end{array} \end{array}$$ (18)

where $\Omega_{j}=1-\frac{B(\frac{n_{j}}{\beta_{j}},2\gamma_{j}-\frac{n}{\beta_{j}})}{2B(\frac{n_{j}}{\beta_{j}},\gamma_{j}-\frac{n_{j}}{\beta_{j}})}$ and *j* = 1, 2. Proof of moment generating function is given in S1 Appendix.

### 3.5 Product moments

If distribution of the random variable (*X*_1_, *X*_2_) is BFGMPLx, then its *r*_1_th and *r*_2_th joint moments around zero denoted by $\mu_{r_{1}r_{2}}^{\prime }$ can be presented as follows:

$$\begin{array}{c} \begin{array}{cccc} \mu_{r_{1}r_{2}}^{\prime }= & \frac{r_{1}}{\beta_{1}}\lambda_{1}^{\frac{r_{1}}{\beta_{1}}}B\left(\frac{r_{1}}{\beta_{1}},\gamma_{1}-\frac{r_{1}}{\beta_{1}}\right)\frac{r_{2}}{\beta_{2}}\lambda_{2}^{\frac{r_{2}}{\beta_{2}}}B\left(\frac{r_{2}}{\beta_{2}},\gamma_{2}-\frac{r_{2}}{\beta_{2}}\right) & & \\ & [1+\theta -2\theta {ϒ}_{2}-2\theta {ϒ}_{1}+4\theta {ϒ}_{1}{ϒ}_{2}], \end{array} \end{array}$$ (19)

where ${ϒ}_{j}=1-\frac{B(\frac{r_{j}}{\beta_{j}},2\gamma_{j}-\frac{r_{j}}{\beta_{j}})}{2B(\frac{r_{j}}{\beta_{j}},\gamma_{j}-\frac{r_{j}}{\beta_{j}})}$ and *j* = 1, 2. Proof the product moments is given in S1 Appendix.

From (45) in S1 Appendix, the covariance and correlation (*ρ*) between *X*_1_ and *X*_2_ are calculated as follows:

$$\begin{array}{c} cov\left(X_{1},X_{2}\right)=\frac{1}{\beta_{1}}\lambda_{1}^{\frac{1}{\beta_{1}}}B\left(\frac{1}{\beta_{1}},\gamma_{1}-\frac{1}{\beta_{1}}\right)\frac{1}{\beta_{2}}\lambda_{2}^{\frac{1}{\beta_{2}}}B\left(\frac{1}{\beta_{2}},\gamma_{2}-\frac{1}{\beta_{2}}\right)\;\theta [1-\Lambda_{1}-\Lambda_{2}+\Lambda_{1}\Lambda_{2}], \end{array}$$ (20)

and

$$\begin{array}{c} \rho \left(X_{1},X_{2}\right)=\frac{\theta (1-\Lambda_{1}-\Lambda_{2}+\Lambda_{1}\Lambda_{2})}{\sqrt{\frac{2\beta_{1}B\left(\frac{2}{\beta_{1}},\gamma_{1}-\frac{2}{\beta_{1}}\right)}{{B(\frac{1}{\beta_{1}},\gamma_{1}-\frac{1}{\beta_{1}})}^{2}}-1}\sqrt{\frac{2\beta_{2}B\left(\frac{2}{\beta_{2}},\gamma_{2}-\frac{2}{\beta_{2}}\right)}{{B(\frac{1}{\beta_{1}},\gamma_{1}-\frac{1}{\beta_{1}})}^{2}}-1}}. \end{array}$$ (21)

We notice that *ρ* = 0 when *θ* = 0, this implies that *X*_1_ and *X*_2_ are independent.

## 4 Positive quadrant dependence

Positive quadrant dependence property of BFGMPLx is presented in this section. Positive quadrant dependence, a type of random variable dependence, was introduced by Lehmann [30]. Two random variables *X*_1_ and *X*_2_ are considered positive quadrant dependent (PQD) if

**Theorem 1**: BFGMPLx is PQD (NQD) for positive (negative) value of *θ*.

**Proof**: Consider

$$\begin{array}{c} Pr\left(X_{1}>x_{1},X_{2}>x_{2}\right)-Pr\left(X_{1}>x_{1}\right)Pr\left(X_{2}>x_{2}\right)=R\left(x_{1},x_{2}\right)-R\left(x_{1}\right)R\left(x_{2}\right), \end{array}$$ (22)

$$\begin{array}{c} \begin{array}{cccc} = & \left[1+\theta (1-\lambda_{1}^{\gamma_{1}}{\left(\lambda_{1}+x_{1}^{\beta_{1}}\right)}^{-\gamma_{1}})(1-\lambda_{2}^{\gamma_{2}}{\left(\lambda_{2}+x_{2}^{\beta_{2}}\right)}^{-\gamma_{2}})][\lambda_{1}^{\gamma_{1}}{\left(\lambda_{1}+x_{1}^{\beta_{1}}\right)}^{-\gamma_{1}}\right] & & \\ & [\lambda_{2}^{\gamma_{2}}{\left(\lambda_{2}+x_{2}^{\beta_{2}}\right)}^{-\gamma_{2}}]-[\lambda_{1}^{\gamma_{1}}{\left(\lambda_{1}+x_{1}^{\beta_{1}}\right)}^{-\gamma_{1}}][\lambda_{2}^{\gamma_{2}}{\left(\lambda_{2}+x_{2}^{\beta_{2}}\right)}^{-\gamma_{2}}], & & \\ = & \theta [\lambda_{1}^{\gamma_{1}}{\left(\lambda_{1}+x_{1}^{\beta_{1}}\right)}^{-\gamma_{1}}][\lambda_{2}^{\gamma_{2}}{\left(\lambda_{2}+x_{2}^{\beta_{2}}\right)}^{-\gamma_{2}}] & & \\ & (1-\lambda_{1}^{\gamma_{1}}{\left(\lambda_{1}+x_{1}^{\beta_{1}}\right)}^{-\gamma_{1}})(1-\lambda_{2}^{\gamma_{2}}{\left(\lambda_{2}+x_{2}^{\beta_{2}}\right)}^{-\gamma_{2}})=\theta \xi \left(x_{1},x_{2}\right), \end{array} \end{array}$$ (23)

where $\xi \left(x_{1},x_{2}\right)=(1-\lambda_{1}^{\gamma_{1}}{\left(\lambda_{1}+x_{1}^{\beta_{1}}\right)}^{-\gamma_{1}})(1-\lambda_{2}^{\gamma_{2}}{\left(\lambda_{2}+x_{2}^{\beta_{2}}\right)}^{-\gamma_{2}})[\lambda_{1}^{\gamma_{1}}{\left(\lambda_{1}+x_{1}^{\beta_{1}}\right)}^{-\gamma_{1}}][\lambda_{2}^{\gamma_{2}}{\left(\lambda_{2}+x_{2}^{\beta_{2}}\right)}^{-\gamma_{2}}]=R\left(x_{1}\right)R\left(x_{2}\right)F_{1}\left(x_{1}\right)F_{2}\left(x_{2}\right)$, which for all values of *x*_1_ and *x*_2_ is always non-negative, because cdf and reliability function takes values ranging from zero to one. As a result, for positive values of *θ*, *θξ*(*x*_1_, *x*_2_) ≥ 0 ∀*x*_1_, *x*_2_. This demonstrates the condition stated in (22). Therefore, BFGMPLx is PQD for positive values of *θ*. Likewise, for negative values of *θ*, *θξ*(*x*_1_, *x*_2_) ≤ 0∀*x*_1_, *x*_2_. As a result, inequality in (22) is reversed, hence for negative values of *θ*, BFGMPLx is NQD. Thus BFGMPLx has both positive and negative quadrant dependence.

## 5 Reliability measures

In this section, we derive reliability measures such as hazard rate, mean residual life, and vitality function in the context of BFGMPLx.

### 5.1 Hazard rate function

Using the definition of bivariate hazard rate function introduced by Basu [31], the hazard rate function of BFGMPLx is obtained as

$$\begin{array}{c} \begin{array}{cccc} h(x_{1},x_{2})= & \frac{\gamma_{1}\beta_{1}x_{1}^{\beta_{1}-1}\gamma_{2}\beta_{2}x_{2}^{\beta_{2}-1}}{\left(\lambda_{1}+x_{1}^{\beta_{1}})(\lambda_{2}+x_{2}^{\beta_{2}}\right)} & & \\ & \frac{\left[1+\theta (2\lambda_{1}^{\gamma_{1}}{\left(\lambda_{1}+x_{1}^{\beta_{1}}\right)}^{-\gamma_{1}}-1)(2\lambda_{2}^{\gamma_{2}}{\left(\lambda_{2}+x_{2}^{\beta_{2}}\right)}^{-\gamma_{2}}-1)\right]}{\left[1+\theta (1-\lambda_{1}^{\gamma_{1}}{\left(\lambda_{1}+x_{1}^{\beta_{1}}\right)}^{-\gamma_{1}})(1-\lambda_{2}^{\gamma_{2}}{\left(\lambda_{2}+x_{2}^{\beta_{2}}\right)}^{-\gamma_{2}})\right]}. \end{array} \end{array}$$ (24)

**Fig 4:**
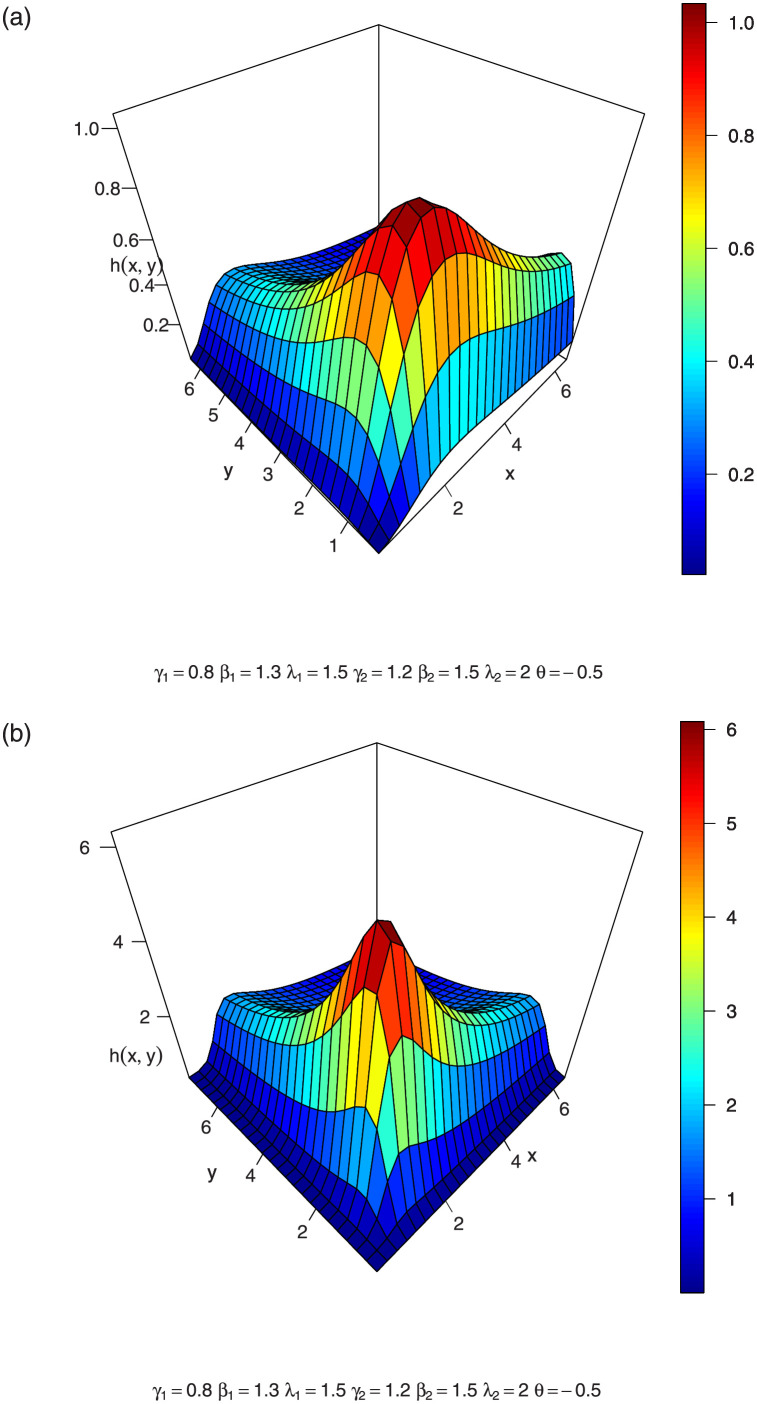
3-dimension of joint hazard of BFGMPLx.

**Fig 5:**
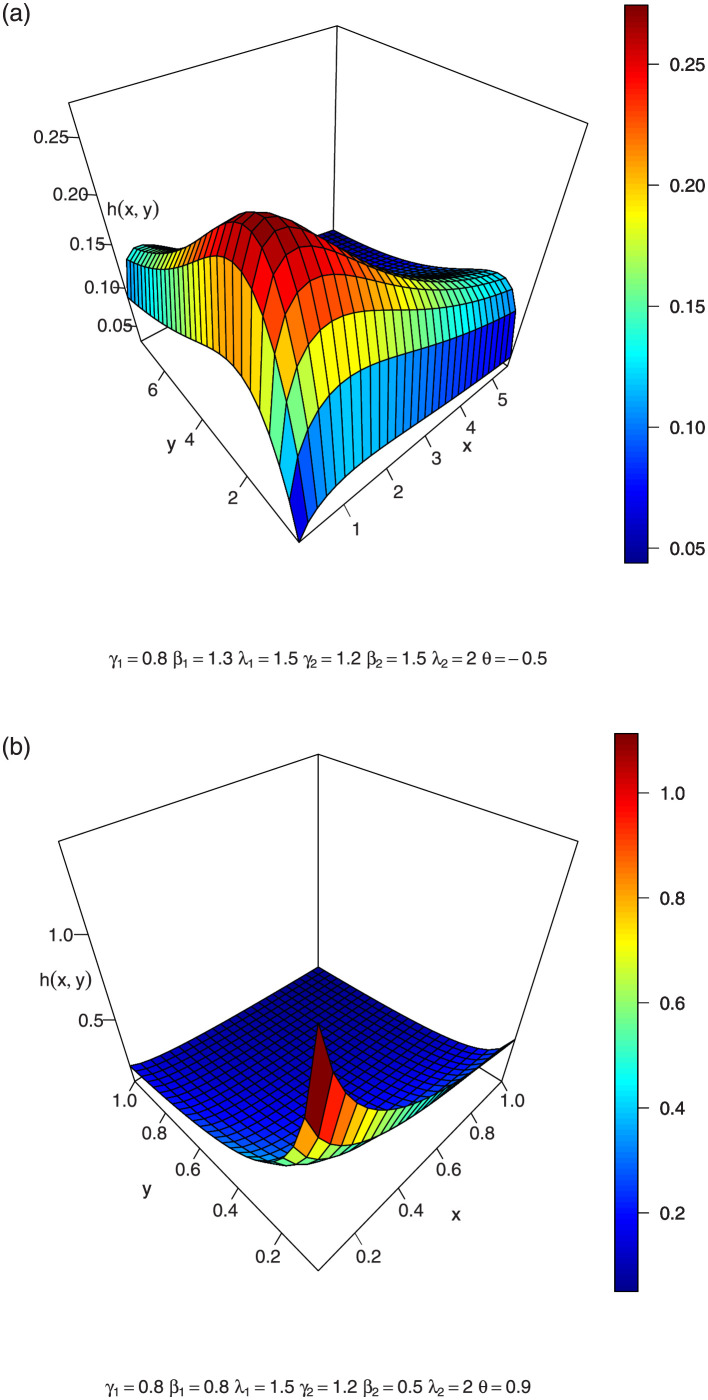
3-dimension of joint hazard of BFGMPLx.

**Fig 6:**
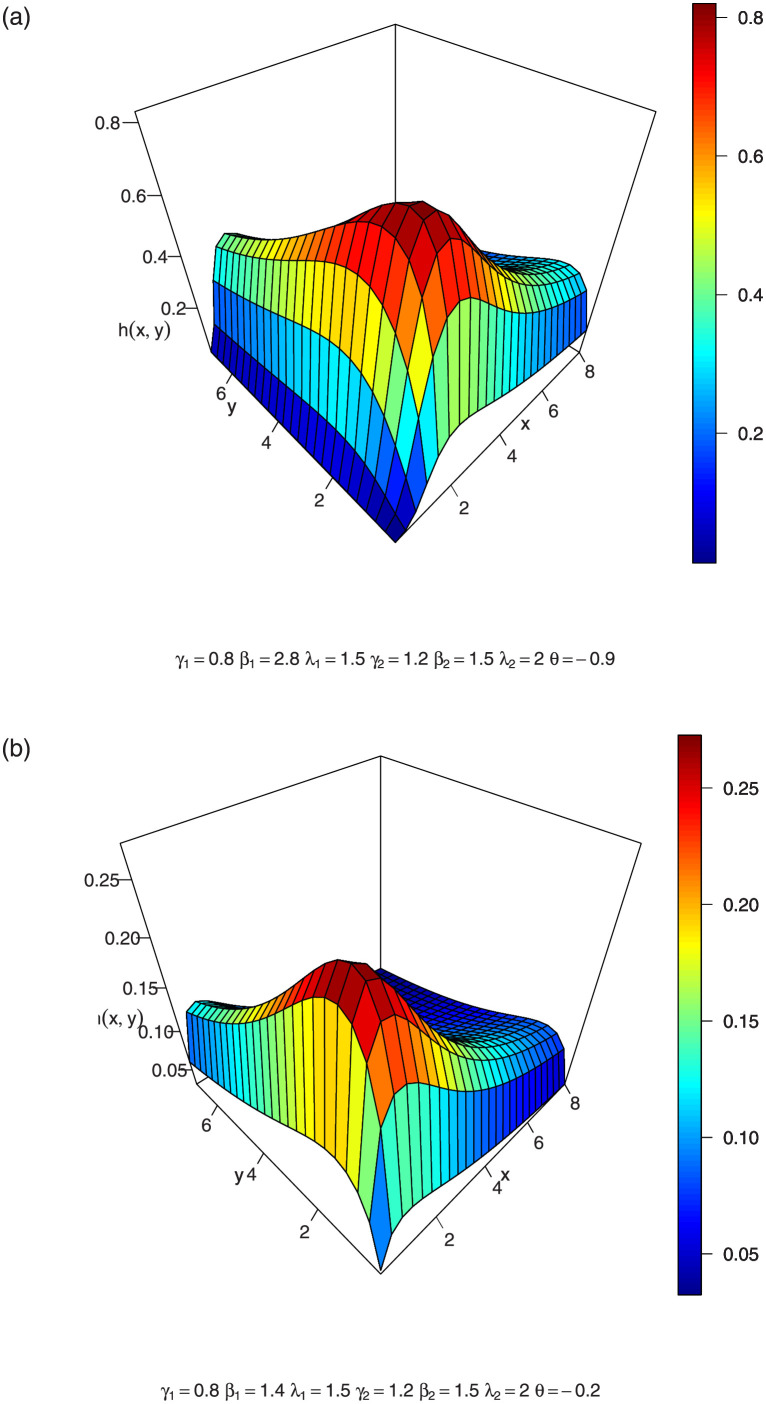
3-dimension of joint hazard of BFGMPLx.

In Figs 4–6, we show the 3D plots of the joint hazard of a BFGMPLx distribution for various parameter values.

Basu has a main constraint, which its definition is from *R*^2^ → *R*, i.e. *h*(*x*_1_, *x*_2_) is not a vector quantity. To overcome this constraint, Johnson et al. [32] and Sreelakshmi [29] introduced the bivariate hazard rate function in vector form as follows:

$$\begin{array}{c} h\left(x_{1},x_{2}\right)=\left(\frac{-\partial \;\text{ln}\;R(x_{1},x_{2})}{\partial x_{1}},\frac{-\partial \;\text{ln}\;R(x_{1},x_{2})}{\partial x_{2}}\right), \end{array}$$ (25)

where *R*(.) denotes the bivariate reliability function. For FGM copula, Vaidyanathan [22] introduced $\frac{-\partial \;\text{ln}\;R(x_{1},x_{2})}{\partial x_{1}}$ as follows:

$$\begin{array}{c} \frac{-\partial \;\text{ln}\;R\left(x_{1},x_{2}\right)}{\partial x_{1}}=h\left(x_{1}\right)[1-{\left({\left[1-F\left(x_{1}\right)\right]}^{-1}[{\left(\theta F\left(x_{2}\right)\right)}^{-1}+1]-1\right)}^{-1}]. \end{array}$$ (26)

From (10), we get

$$\begin{array}{c} \begin{array}{cccc} \frac{-\partial \;\text{ln}\;R\left(x_{1},x_{2}\right)}{\partial x_{1}} & =\frac{\gamma_{1}\beta_{1}x_{1}^{\beta_{1}-1}}{\lambda_{1}+x_{1}^{\beta_{1}}} & & \\ & [1-A^{-1}\lambda_{1}^{\gamma_{1}}\theta \left(1-\lambda_{2}^{\gamma_{2}}{\left(\lambda_{2}+x_{2}^{\beta_{2}}\right)}^{-\gamma_{2}}\right)\left({\left(\lambda_{1}+x_{1}^{\beta_{1}}\right)}^{-\gamma_{1}}\right)], \end{array} \end{array}$$ (27)

$$\begin{array}{c} \begin{array}{cccc} \frac{-\partial \;\text{ln}\;R\left(x_{1},x_{2}\right)}{\partial x_{2}} & =\frac{\gamma_{2}\beta_{2}x_{2}^{\beta_{2}-1}}{\lambda_{2}+x_{2}^{\beta_{2}}} & & \\ & [1-A^{-1}\lambda_{2}^{\gamma_{2}}\theta \left(1-\lambda_{1}^{\gamma_{1}}{\left(\lambda_{1}+x_{1}^{\beta_{1}}\right)}^{-\gamma_{1}}\right)\left({\left(\lambda_{2}+x_{2}^{\beta_{2}}\right)}^{-\gamma_{2}}\right)], \end{array} \end{array}$$ (28)

where $A=[1+\theta \left(1-\lambda_{1}^{\gamma_{1}}{\left(\lambda_{1}+x_{1}^{\beta_{1}}\right)}^{-\gamma_{1}}\right)\left(1-\lambda_{2}^{\gamma_{2}}{\left(\lambda_{2}+x_{2}^{\beta_{2}}\right)}^{-\gamma_{2}}\right)]$.

**Fig 7:**
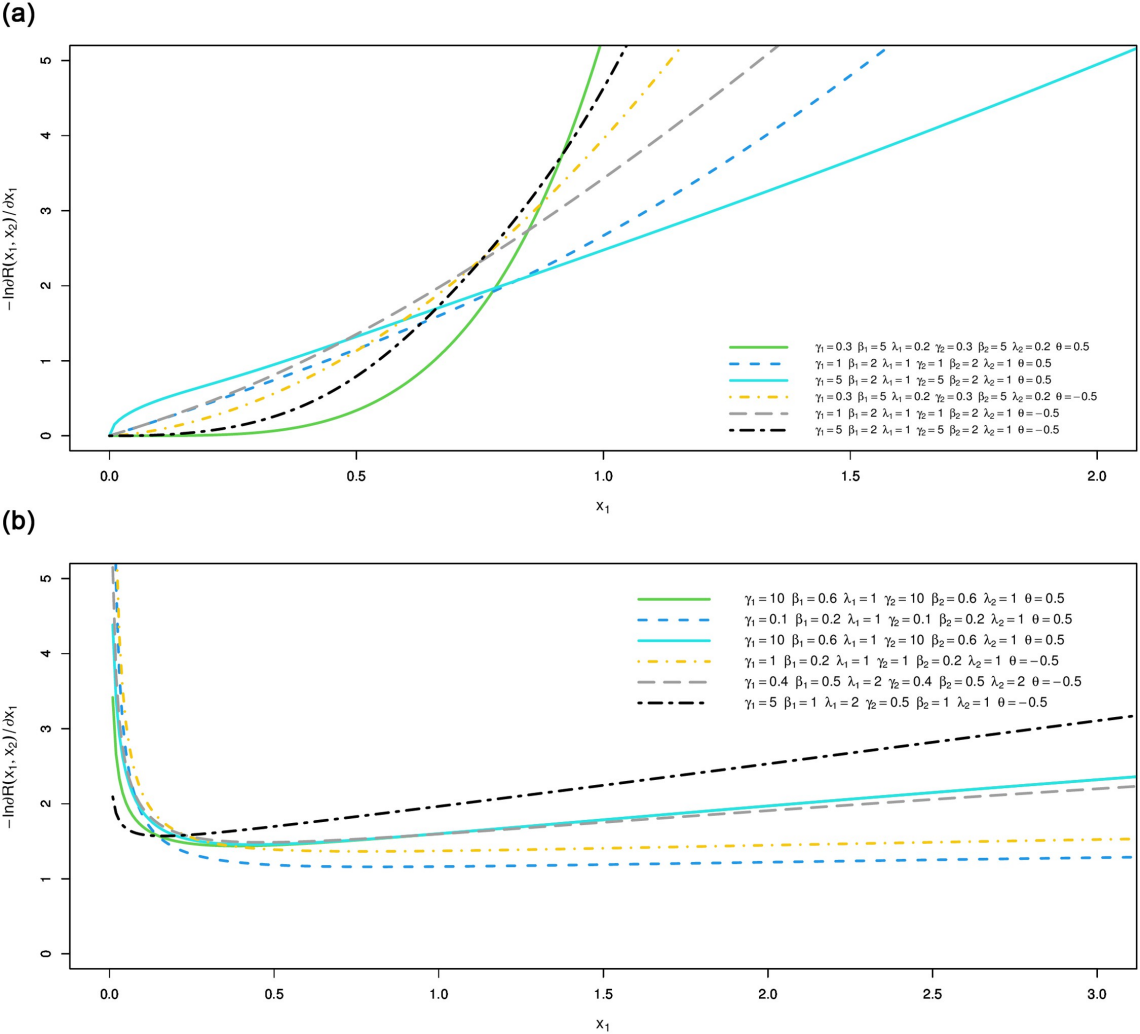
Possible shapes of the vector of the hrf for the BFGMPLx depending on *x*_1_ for several values of parameters.

The vector hazard rate function of BFGMPLx is obtained by substituting the above expressions in (25). Eq (27) consists of two terms: the first term $\frac{\gamma_{1}\beta_{1}x_{1}^{\beta_{1}-1}}{\lambda_{1}+x_{1}^{\beta_{1}}}$ is the hazard rate of the power lomax distribution, which is an inverted bathtub (IBT) for *β* > 1 and is a decreasing hazard rate (DHR) for 0 < *β* ≤ 1, according to [6]. The second term $\left[1-A^{-1}\lambda_{1}^{\gamma_{1}}\theta \left(1-\lambda_{2}^{\gamma_{2}}{\left(\lambda_{2}+x_{2}^{\beta_{2}}\right)}^{-\gamma_{2}}\right)\left({\left(\lambda_{1}+x_{1}^{\beta_{1}}\right)}^{-\gamma_{1}}\right)\right]$ is a positive increasing function for positive *θ* and is a negative decreasing function for negative *θ*. Thus, for positive and negative values of *θ*, BFGMPLx is J-HR and IHR for *β* > 1 and is Reversed J-HR and Bathtub for 0 < *β* ≤ 1. Eq (28) exhibits similar behavior. Fig 7 shows the shapes of the vector hazard rate function of BFGMPLx depending on *x*_1_ for several values of parameters.

### 5.2 Mean residual life

The average remaining life of a unit after it has survived for a particular time *t* is denoted by mean residual life (MRL). Shanbag and Kotz [33] defined MRL for vector-valued random variables as:

$$\begin{array}{c} m\left(x_{1},x_{2}\right)=\left(m_{1}\left(x_{1},x_{2}\right),m_{2}\left(x_{1},x_{2}\right)\right), \end{array}$$ (29)

where

$$\begin{array}{c} m_{1}\left(x_{1},x_{2}\right)=E\left(X_{1}-x_{1}|X_{1}\geq x_{1},X_{2}\geq x_{2}\right), \end{array}$$

and

$$\begin{array}{c} m_{2}\left(x_{1},x_{2}\right)=E\left(X_{2}-x_{2}|X_{1}\geq x_{1},X_{2}\geq x_{2}\right). \end{array}$$

The expressions for *m*_1_(*x*_1_, *x*_2_) and *m*_2_(*x*_1_, *x*_2_) in BFGMPLx are obtained as:

$$\begin{array}{c} m_{1}\left(x_{1},x_{2}\right)=\frac{\frac{\lambda_{1}^{\frac{1}{\beta_{1}}}}{\beta_{1}}B\left(\frac{1}{\beta_{1}},\gamma_{1}-\frac{1}{\beta_{1}}\right)[1+\theta \left(1-\Lambda_{1}\right)\left(1-\lambda_{2}^{\gamma_{2}}{\left(\lambda_{2}+x_{2}^{\beta_{2}}\right)}^{-\gamma_{2}}\right)]}{[\lambda_{1}^{\gamma_{1}}{\left(\lambda_{1}+x_{1}^{\beta_{1}}\right)}^{-\gamma_{1}}]A}, \end{array}$$ (30)

$$\begin{array}{c} m_{2}\left(x_{1},x_{2}\right)=\frac{\frac{\lambda_{2}^{\frac{1}{\beta_{2}}}}{\beta_{2}}B\left(\frac{1}{\beta_{2}},\gamma_{2}-\frac{1}{\beta_{2}}\right)[1+\theta \left(1-\Lambda_{2}\right)\left(1-\lambda_{1}^{\gamma_{1}}{\left(\lambda_{1}+x_{1}^{\beta_{1}}\right)}^{-\gamma_{1}}\right)]}{[\lambda_{2}^{\gamma_{2}}{\left(\lambda_{2}+x_{2}^{\beta_{2}}\right)}^{-\gamma_{2}}]A}. \end{array}$$ (31)

Substituting (30) and (31) in (29) yields BFGMPLx’s MRL.

### 5.3 Vitality function

Sankaran and Nair [34] defined a bivariate vitality function for a two-component system as:

$$\begin{array}{c} \upsilon \left(x_{1},x_{2}\right)=\left(\upsilon_{1}\left(x_{1},x_{2}\right),\upsilon_{2}\left(x_{1},x_{2}\right)\right), \end{array}$$ (32)

where

$$\begin{array}{c} \upsilon_{1}\left(x_{1},x_{2}\right)=E\left(X_{1}|X_{1}\geq x_{1},X_{2}\geq x_{2}\right), \end{array}$$

and

$$\begin{array}{c} \upsilon_{2}\left(x_{1},x_{2}\right)=E\left(X_{2}|X_{1}\geq x_{1},X_{2}\geq x_{2}\right). \end{array}$$

Also, *υ*_*i*_(*x*_1_, *x*_2_) is related to *m*_*i*_(*x*_1_, *x*_2_) by

$$\begin{array}{c} \upsilon_{i}\left(x_{1},x_{2}\right)=x_{i}+m_{i}\left(x_{1},x_{2}\right),\;i=1,2. \end{array}$$ (33)

Here *υ*_1_(*x*_1_, *x*_2_) calculates the expected life time of first component as the sum of current age *x*_1_ and the average lifetime remaining to it, assuming the second component has survived past age *x*_2_. *υ*_2_(*x*_1_, *x*_2_) has a similar interpretation. Using Eqs (30) and (31) in (33), we obtain *υ*_1_(*x*_1_, *x*_2_) and *υ*_2_(*x*_1_, *x*_2_) of BFGMPLx as:

$$\begin{array}{c} \upsilon_{1}\left(x_{1},x_{2}\right)=x_{1}+\frac{\frac{\lambda_{1}^{\frac{1}{\beta_{1}}}}{\beta_{1}}B\left(\frac{1}{\beta_{1}},\gamma_{1}-\frac{1}{\beta_{1}}\right)[1+\theta \left(1-\Lambda_{1}\right)\left(1-\lambda_{2}^{\gamma_{2}}{\left(\lambda_{2}+x_{2}^{\beta_{2}}\right)}^{-\gamma_{2}}\right)]}{[\lambda_{1}^{\gamma_{1}}{\left(\lambda_{1}+x_{1}^{\beta_{1}}\right)}^{-\gamma_{1}}]A}, \end{array}$$ (34)

$$\begin{array}{c} \upsilon_{2}\left(x_{1},x_{2}\right)=x_{2}+\frac{\frac{\lambda_{2}^{\frac{1}{\beta_{2}}}}{\beta_{2}}B\left(\frac{1}{\beta_{2}},\gamma_{2}-\frac{1}{\beta_{2}}\right)[1+\theta \left(1-\Lambda_{2}\right)\left(1-\lambda_{1}^{\gamma_{1}}{\left(\lambda_{1}+x_{1}^{\beta_{1}}\right)}^{-\gamma_{1}}\right)]}{[\lambda_{2}^{\gamma_{2}}{\left(\lambda_{2}+x_{2}^{\beta_{2}}\right)}^{-\gamma_{2}}]A} \end{array}$$ (35)

From (34) and (35), the vitality function of BFGMPLx can be obtained using (32).

## 6 Methods of estimation

In this section, we present two estimation methods for estimating the unknown parameters of the BFGMPLx distribution: maximum likelihood estimation (MLE) and Bayesian estimation.

### 6.1 Maximum likelihood estimation

Let (*x*_*i*1_, *x*_*i*2_), *i* = 1, 2, …, *n* denote random samples from BFGMPLx with parameters Θ = (*β*_1_, *γ*_1_, λ_1_, *β*_2_, *γ*_2_, λ_2_, *θ*). the log likelihood function lnL is obtained by using the density function given in (8),

$$\begin{array}{c} \begin{array}{cccc} \;\text{ln}\;L= & n\;\text{ln}\;\gamma_{1}+n\;\text{ln}\;\beta_{1}+n\gamma_{1}\;\text{ln}\;\lambda_{1}+\left(\beta_{1}-1\right)\sum_{i=1}^{n}\;\text{ln}\;\left(x_{i1}\right) & & \\ & -\left(\gamma_{1}+1\right)\sum_{i=1}^{n}\;\text{ln}\;\left(\lambda_{1}+x_{i1}^{\beta_{1}}\right)+n\;\text{ln}\;\gamma_{2}+n\;\text{ln}\;\beta_{2}+n\gamma_{2}\;\text{ln}\;\lambda_{2} & & \\ & +\left(\beta_{2}-1\right)\sum_{i=1}^{n}\;\text{ln}\;\left(x_{i2}\right)-\left(\gamma_{2}+1\right)\sum_{i=1}^{n}\;\text{ln}\;\left(\lambda_{2}+x_{i2}^{\beta_{2}}\right) & & \\ & +\sum_{i=1}^{n}\;\text{ln}\;[1+\theta \left(2\lambda_{1}^{\gamma_{1}}{\left(\lambda_{1}+x_{i1}^{\beta_{1}}\right)}^{-\gamma_{1}}-1\right)\left(2\lambda_{2}^{\gamma_{2}}{\left(\lambda_{2}+x_{i2}^{\beta_{2}}\right)}^{-\gamma_{2}}-1\right)], \end{array} \end{array}$$ (36)

We obtain the following likelihood equations by partially differentiating *lnL* with respect to the vector of parameters Θ and equating them to zero. The following are the first derivatives:

$$\begin{array}{c} \begin{array}{cccc} \frac{\partial L\left(\Theta \right)}{\partial \gamma_{l}}= & \frac{n}{\gamma_{l}}+n\;\text{ln}\;\lambda_{l}-\sum_{i=1}^{n}\;\text{ln}\;\left(\lambda_{l}+x_{il}^{\beta_{l}}\right) & & \\ & +\sum_{i=1}^{n}\frac{2\theta \lambda_{l}^{\gamma_{l}}{\left(\lambda_{l}+x_{il}^{\beta_{l}}\right)}^{-\gamma_{l}}\left(2\lambda_{j}^{\gamma_{j}}{\left(\lambda_{j}+x_{ij}^{\beta_{j}}\right)}^{-\gamma_{j}}-1\right)\left(\;\text{ln}\;\lambda_{l}-\;\text{ln}\;\left(\lambda_{l}+x_{il}^{\beta_{l}}\right)\right)}{1+\theta \left(2\lambda_{1}^{\gamma_{1}}{\left(\lambda_{1}+x_{i1}^{\beta_{1}}\right)}^{-\gamma_{1}}-1\right)\left(2\lambda_{2}^{\gamma_{2}}{\left(\lambda_{2}+x_{i2}^{\beta_{2}}\right)}^{-\gamma_{2}}-1\right)}, \end{array} \end{array}$$ (37)

$$\begin{array}{c} \begin{array}{cccc} \frac{\partial L\left(\Theta \right)}{\partial \beta_{l}}= & \frac{n}{\beta_{l}}+\sum_{i=1}^{n}\;\text{ln}\;\left(x_{il}\right)-\sum_{i=1}^{n}\frac{\left(\gamma_{l}+1\right)x_{il}^{\beta_{l}}\;\text{ln}\;\left(x_{il}\right)}{\left(\lambda_{l}+x_{il}^{\beta_{l}}\right)} & & \\ & -\sum_{i=1}^{n}\frac{2\theta \gamma_{l}\lambda_{l}^{\gamma_{l}}x_{il}^{\beta_{l}}\;\text{ln}\;\left(x_{il}\right){\left(\lambda_{l}+x_{il}^{\beta_{l}}\right)}^{-\gamma_{l}-1}\left(2\lambda_{j}^{\gamma_{j}}{\left(\lambda_{j}+x_{ij}^{\beta_{j}}\right)}^{-\gamma_{j}}-1\right)}{1+\theta \left(2\lambda_{1}^{\gamma_{1}}{\left(\lambda_{1}+x_{i1}^{\beta_{1}}\right)}^{-\gamma_{1}}-1\right)\left(2\lambda_{2}^{\gamma_{2}}{\left(\lambda_{2}+x_{i2}^{\beta_{2}}\right)}^{-\gamma_{2}}-1\right)}, & \end{array} \end{array}$$ (38)

$$\begin{array}{c} \begin{array}{cccc} \frac{\partial L\left(\Theta \right)}{\partial \lambda_{l}}= & \frac{n\gamma_{l}}{\lambda_{l}}-\sum_{i=1}^{n}\frac{\gamma_{l}+1}{\left(\lambda_{l}+x_{il}^{\beta_{l}}\right)} & & \\ & +\sum_{i=1}^{n}\frac{2\theta \gamma_{l}\lambda_{l}^{\gamma_{l}}{\left(\lambda_{l}+x_{il}^{\beta_{l}}\right)}^{-\gamma_{l}}\left(2\lambda_{j}^{\gamma_{j}}{\left(\lambda_{j}+x_{ij}^{\beta_{j}}\right)}^{-\gamma_{j}}-1\right)\left(\frac{1}{\lambda_{l}}-\frac{1}{\left(\lambda_{l}+x_{il}^{\beta_{l}}\right)}\right)}{1+\theta \left(2\lambda_{1}^{\gamma_{1}}{\left(\lambda_{1}+x_{i1}^{\beta_{1}}\right)}^{-\gamma_{1}}-1\right)\left(2\lambda_{2}^{\gamma_{2}}{\left(\lambda_{2}+x_{i2}^{\beta_{2}}\right)}^{-\gamma_{2}}-1\right)}, & \end{array} \end{array}$$ (39)

$$\begin{array}{c} \frac{\partial L\left(\Theta \right)}{\partial \theta }=\sum_{i=1}^{n}\frac{\left(2\lambda_{1}^{\gamma_{1}}{\left(\lambda_{1}+x_{i1}^{\beta_{1}}\right)}^{-\gamma_{1}}-1\right)\left(2\lambda_{2}^{\gamma_{2}}{\left(\lambda_{2}+x_{i2}^{\beta_{2}}\right)}^{-\gamma_{2}}-1\right)}{1+\theta \left(2\lambda_{1}^{\gamma_{1}}{\left(\lambda_{1}+x_{i1}^{\beta_{1}}\right)}^{-\gamma_{1}}-1\right)\left(2\lambda_{2}^{\gamma_{2}}{\left(\lambda_{2}+x_{i2}^{\beta_{2}}\right)}^{-\gamma_{2}}-1\right)}, \end{array}$$ (40)

where *j* = (1, 2), *l* = (1, 2); *j* ≠ *l*, (for example *j* = 1 then *l* = 2).

### 6.2 Bayesian estimation

Here, we employ the symmetric loss functions to derive the Bayes estimators of the BFGMPLx distribution’s parameters. We must select an acceptable prior density function and hyper-parameter values that reflect our belief regarding the data. We use the symmetric square error loss function (SSELF) to get the estimates based on a complete sample and assume that the parameters of BFGMPLx distribution are independent. For the parameters *γ*_*l*_, *β*_*l*_, and λ_*l*_, we select gamma-independent priors, specifically,

$$\begin{array}{c} \pi_{1}\left(\gamma_{l}\right)\propto \gamma_{l}^{q_{l}-1}e^{-w_{l}\gamma_{l}},\;\gamma_{l}>0,q_{l},w_{l}>0,\\ \pi_{2}\left(\beta_{l}\right)\propto \beta_{l}^{r_{l}-1}e^{-u_{l}\beta_{l}},\;\beta_{l}>0,r_{l},u_{l}>0,\\ \pi_{3}\left(\lambda_{l}\right)\propto \lambda_{l}^{o_{l}-1}e^{-p_{l}\lambda_{l}},\;\lambda_{l}>0,o_{l},p_{l}>0, \end{array}$$

while the copula parameter *θ* has uniform prior distribution where −1 < *θ* < 1 The joint prior Eq (41) as follows:

$$\begin{array}{cc} \pi \left(\Theta \right) & \propto \gamma_{1}^{q_{1}-1}e^{-w_{1}\gamma_{1}}\beta_{1}^{r_{1}-1}e^{-u_{1}\beta_{1}}\lambda_{1}^{o_{1}-1}e^{-p_{1}\lambda_{1}}\gamma_{2}^{q_{2}-1}e^{-w_{2}\gamma_{2}}\beta_{2}^{r_{2}-1}e^{-u_{2}\beta_{2}}\lambda_{2}^{o_{2}-1}e^{-p_{2}\lambda_{2}}, \end{array}$$ (41)

The likelihood method’s estimate and variance-covariance matrix can be used to determine how to elicit the independent joint prior’s hyper-parameters. Gamma priors’ mean and variance can be used to represent the derived hyper-parameters. For more information see [35–37]. The parameters *γ*_*l*_, *β*_*l*_, and λ_*l*_ where *l* = 1, 2, of BFGMPLx distribution should be well-known and positive. The likelihood function, Eq (43) is as follows:

$$\begin{array}{c} \begin{array}{cccc} L\left(\Theta \right)= & \gamma_{1}^{n}\beta_{1}^{n}\lambda_{1}^{n\gamma_{1}}\prod_{i=1}^{n}x_{i1}^{\beta_{1}-1}{\left(\lambda_{1}+x_{i1}^{\beta_{1}}\right)}^{-\gamma_{1}-1}\gamma_{2}^{n}\beta_{2}^{n}\lambda_{2}^{n\gamma_{2}}\prod_{i=1}^{n}x_{i2}^{\beta_{2}-1}{\left(\lambda_{2}+x_{i2}^{\beta_{2}}\right)}^{-\gamma_{2}-1} & & \\ & \prod_{i=1}^{n}[1+\theta \left(2\lambda_{1}^{\gamma_{1}}{\left(\lambda_{1}+x_{i1}^{\beta_{1}}\right)}^{-\gamma_{1}}-1\right)\left(2\lambda_{2}^{\gamma_{2}}{\left(\lambda_{2}+x_{i2}^{\beta_{2}}\right)}^{-\gamma_{2}}-1\right)], \end{array} \end{array}$$ (42)

and the joint posterior distribution can be expressed using the joint prior function Eq (41) and likelihood function Eq (43). Consequently, the function of the Θ joint’s posterior density is

$$\begin{array}{c} \begin{array}{cccc} \Pi \left(\Theta |x_{1},x_{2}\right)\propto & \gamma_{1}^{n+q_{1}-1}\beta_{1}^{n+r_{1}-1}\lambda_{1}^{n\gamma_{1}+o_{1}-1}e^{-w_{1}\gamma_{1}-u_{1}\beta_{1}-p_{1}\lambda_{1}}\gamma_{2}^{n+q_{2}-1} & & \\ & \prod_{i=1}^{n}x_{i1}^{\beta_{1}-1}{\left(\lambda_{1}+x_{i1}^{\beta_{1}}\right)}^{-\gamma_{1}-1}\prod_{i=1}^{n}x_{i2}^{\beta_{2}-1}{\left(\lambda_{2}+x_{i2}^{\beta_{2}}\right)}^{-\gamma_{2}-1} & & \\ & e^{-w_{2}\gamma_{2}-u_{2}\beta_{2}-p_{2}\lambda_{2}}\beta_{2}^{n+r_{2}-1}\lambda_{2}^{n\gamma_{2}+o_{2}-1} & & \\ & \prod_{i=1}^{n}[1+\theta \left(2\lambda_{1}^{\gamma_{1}}{\left(\lambda_{1}+x_{i1}^{\beta_{1}}\right)}^{-\gamma_{1}}-1\right)\left(2\lambda_{2}^{\gamma_{2}}{\left(\lambda_{2}+x_{i2}^{\beta_{2}}\right)}^{-\gamma_{2}}-1\right)]. \end{array} \end{array}$$ (43)

The symmetric loss function is the squared-error loss function, abbreviated as SELF. The Bayesian estimator of Θ under SELF is then the average $\tilde{\Theta }=E_{\Theta }\left(\Theta \right)$.

The expectation of loss functions are difficult to analyse by mathematical integration, hence the Markov Chain Monte Carlo (MCMC) approach will be utilised. Gibbs sampling and the broader Metropolis-within-Gibbs samplers are the two most significant MCMC algorithm sub-classes. Robert et al. [38] covered this algorithm. Similar to acceptance-rejection sampling, the Metropolis-Hastings (MH) method treats a candidate value derived from a proposal distribution as normal for each iteration of the process. The MH approach computes an appropriate transition in two phases, starting with $\Theta_{i}={\hat{\Theta }}_{i}$:

- Take *π*(Θ*|Θ) from a proposal distribution while Θ* is a constant.
- You can either stick with the current sample Θ_*i*+1_ = Θ or switch to Θ_*i*+1_ = Θ*. with acceptance likelihood.
  $$\begin{array}{c} a_{\Theta^{*}|\Theta }=min[1,\;\frac{G\left(\Theta^{*}|x\right)\;\pi \left(\Theta \right)}{G\left(\Theta |x\right)\;\pi \left(\Theta^{*}|\Theta \right)}]. \end{array}$$

In addition to ensuring that the goal density stays invariant, Θ, this well-stated transition density guarantees that the chain converges to its specific invariant density starting from any initial condition.

Using the posterior conditional density functions of the relevant parameters, this method’s basic idea is to provide posterior samples of the relevant parameters. The posterior density function of the relevant parameters is provided by Eq. (5.3). The posterior conditional density functions of *γ*_*l*_, *β*_*l*_, and λ_*l*_ where *l* = 1, 2 can be constructed as follows from this equation:

$$\begin{array}{c} \begin{array}{cccc} \Pi \left(\gamma_{l}|\beta_{l},\lambda_{l},\theta ,x_{1},x_{2}\right)\propto & \gamma_{l}^{n+q_{l}-1}\lambda_{l}^{n\gamma_{l}}e^{-w_{l}\gamma_{l}}\prod_{i=1}^{n}{\left(\lambda_{l}+x_{il}^{\beta_{l}}\right)}^{-\gamma_{l}-1} & & \\ & [1+\theta \left(2\lambda_{1}^{\gamma_{1}}{\left(\lambda_{1}+x_{i1}^{\beta_{1}}\right)}^{-\gamma_{1}}-1\right)\left(2\lambda_{2}^{\gamma_{2}}{\left(\lambda_{2}+x_{i2}^{\beta_{2}}\right)}^{-\gamma_{2}}-1\right)], \end{array} \end{array}$$

$$\begin{array}{c} \begin{array}{cccc} \Pi \left(\beta_{l}|\gamma_{l},\lambda_{l},\theta ,x_{1},x_{2}\right)\propto & \beta_{l}^{n+r_{l}-1}e^{-u_{l}\beta_{l}}\prod_{i=1}^{n}x_{il}^{\beta_{l}-1}{\left(\lambda_{l}+x_{il}^{\beta_{l}}\right)}^{-\gamma_{l}-1} & & \\ & [1+\theta \left(2\lambda_{1}^{\gamma_{1}}{\left(\lambda_{1}+x_{i1}^{\beta_{1}}\right)}^{-\gamma_{1}}-1\right)\left(2\lambda_{2}^{\gamma_{2}}{\left(\lambda_{2}+x_{i2}^{\beta_{2}}\right)}^{-\gamma_{2}}-1\right)], \end{array} \end{array}$$

$$\begin{array}{c} \begin{array}{cccc} \Pi \left(\lambda_{l}|\gamma_{l},\beta_{l},\theta ,x_{1},x_{2}\right) & \propto \lambda_{l}^{n\gamma_{l}+o_{l}-1}e^{-p_{l}\lambda_{l}}\prod_{i=1}^{n}{\left(\lambda_{l}+x_{il}^{\beta_{l}}\right)}^{-\gamma_{l}-1} & & \\ & [1+\theta \left(2\lambda_{1}^{\gamma_{1}}{\left(\lambda_{1}+x_{i1}^{\beta_{1}}\right)}^{-\gamma_{1}}-1\right)\left(2\lambda_{2}^{\gamma_{2}}{\left(\lambda_{2}+x_{i2}^{\beta_{2}}\right)}^{-\gamma_{2}}-1\right)], \end{array} \end{array}$$

$$\begin{array}{c} \begin{array}{cc} \Pi \left(\theta |\gamma_{l},\beta_{l},\lambda_{l},x_{1},x_{2}\right)\propto & \;\prod_{i=1}^{n}[1+\theta \left(2\lambda_{1}^{\gamma_{1}}{\left(\lambda_{1}+x_{i1}^{\beta_{1}}\right)}^{-\gamma_{1}}-1\right)\left(2\lambda_{2}^{\gamma_{2}}{\left(\lambda_{2}+x_{i2}^{\beta_{2}}\right)}^{-\gamma_{2}}-1\right)], \end{array} \end{array}$$

where *l* is 1 and 2.

## 7 Confidence intervals

In this section, we present two different methods to construct confidence intervals (CI) for the unknown parameters of BFGMPLx distribution, which are asymptotic confidence intervals (ACI) and highest posterior density Bayesian estimation.

### 7.1 Asymptotic confidence intervals

The asymptotic normal distribution of the MLE is the most widely used technique for establishing confidence bounds for the parameters. With respect to the Fisher information matrix *I*(Θ), which is comprised of the negative second derivatives of the natural logarithm of the likelihood function evaluated at $\hat{\Theta }=\left(\hat{\gamma_{1}},\hat{\beta_{1}},\hat{\lambda_{1}},\hat{\gamma_{2}},\hat{\beta_{2}},\hat{\lambda_{2}},\hat{\theta }\right)$, the asymptotic variance-covariance matrix of the MLE of the parameters, suppose that the asymptotic variance-covariance matrix of the parameter vector is

$$\begin{array}{c} I\left(\hat{\Theta }\right)=-E[\begin{array}{ccccccc} I_{\hat{\gamma_{1}}\hat{\gamma_{1}}} & & & & & & \\ I_{\hat{\beta_{1}}\hat{\gamma_{1}}} & I_{\hat{\beta_{1}}\hat{\beta_{1}}} & & & & & \\ I_{\hat{\lambda_{1}}\hat{\gamma_{1}}} & I_{\hat{\lambda_{1}}\hat{\beta_{1}}} & I_{\hat{\lambda_{1}}\hat{\lambda_{1}}} & & & & \\ I_{\hat{\gamma_{2}}\hat{\gamma_{1}}} & I_{\hat{\gamma_{2}}\hat{\beta_{1}}} & I_{\hat{\gamma_{2}}\hat{\lambda_{1}}} & I_{\hat{\gamma_{2}}\hat{\gamma_{2}}} & & & \\ I_{\hat{\beta_{2}}\hat{\gamma_{1}}} & I_{\hat{\beta_{2}}\hat{\beta_{1}}} & I_{\hat{\beta_{2}}\hat{\lambda_{1}}} & I_{\hat{\beta_{2}}\hat{\gamma_{2}}} & I_{\hat{\beta_{2}}\hat{\beta_{2}}} & & \\ I_{\hat{\lambda_{2}}\hat{\gamma_{1}}} & I_{\hat{\lambda_{2}}\hat{\beta_{1}}} & I_{\hat{\lambda_{2}}\hat{\lambda_{1}}} & I_{\hat{\lambda_{2}}\hat{\gamma_{2}}} & I_{\hat{\lambda_{2}}\hat{\beta_{2}}} & I_{\hat{\lambda_{2}}\hat{\lambda_{2}}} & \\ I_{\hat{\theta }\hat{\gamma_{1}}} & I_{\hat{\theta }\hat{\beta_{1}}} & I_{\hat{\theta }\hat{\lambda_{1}}} & I_{\hat{\theta }\hat{\gamma_{2}}} & I_{\hat{\theta }\hat{\beta_{2}}} & I_{\hat{\theta }\hat{\lambda_{2}}} & I_{\hat{\theta }\hat{\theta }} \end{array}] \end{array}$$ (44)

where $V\left(\hat{\Theta }\right)=I^{-1}\left(\hat{\Theta }\right)$. Based on the asymptotic normality of the MLE, a 100(1 − *α*)% confidence interval for parameter Θ can be constructed as: $\hat{\gamma_{l}}\pm Z_{0.025}\sqrt{I_{\hat{\gamma_{1}}\hat{\gamma_{1}}}}$, $\hat{\beta_{l}}\pm Z_{0.025}\sqrt{I_{\hat{\beta_{l}}\hat{\beta_{l}}}}$, $\hat{\lambda_{l}}\pm Z_{0.025}\sqrt{I_{\hat{\lambda_{l}}\hat{\lambda_{l}}}}$ and $\hat{\theta }\pm Z_{0.025}\sqrt{I_{\hat{\theta }\hat{\theta }}}$, where *l* = 1, 2 and *Z*_0.025_ is the percentile of the standard normal distribution with right tail probability $\frac{\alpha }{2}$. The second derivatives of the likelihood function with respect to the parameters are as follows

$$\begin{array}{c} \begin{array}{cccc} I_{\gamma_{l}\gamma_{l}}= & \frac{\partial^{2}L\left(\Theta \right)}{\partial \gamma_{l}^{2}}=\frac{-n}{\gamma_{l}^{2}}+\sum_{i=1}^{n}\{2\theta \lambda_{l}^{\gamma_{l}}{\left(\lambda_{l}+x_{il}^{\beta_{l}}\right)}^{-\gamma_{l}}\\ & \frac{\left(2\lambda_{j}^{\gamma_{j}}{\left(\lambda_{j}+x_{ij}^{\beta_{j}}\right)}^{-\gamma_{j}}-1\right)\left({\left(\;\text{ln}\;\lambda_{l}\right)}^{2}-{\left(\;\text{ln}\;\left(\lambda_{l}+x_{il}^{\beta_{l}}\right)\right)}^{2}\right)}{1+\theta \left(2\lambda_{1}^{\gamma_{1}}{\left(\lambda_{1}+x_{i1}^{\beta_{1}}\right)}^{-\gamma_{1}}-1\right)\left(2\lambda_{2}^{\gamma_{2}}{\left(\lambda_{2}+x_{i2}^{\beta_{2}}\right)}^{-\gamma_{2}}-1\right)}\} & & \\ & -\sum_{i=1}^{n}\{4\theta^{2}\lambda_{l}^{2\gamma_{l}}{\left(\lambda_{l}+x_{il}^{\beta_{l}}\right)}^{-2\gamma_{l}}\\ & \;\;\;\frac{{\left(2\lambda_{j}^{\gamma_{j}}{\left(\lambda_{j}+x_{ij}^{\beta_{j}}\right)}^{-\gamma_{j}}-1\right)}^{2}{\left(\;\text{ln}\;\lambda_{l}-\;\text{ln}\;\left(\lambda_{l}+x_{il}^{\beta_{l}}\right)\right)}^{2}}{{\left[1+\theta \left(2\lambda_{1}^{\gamma_{1}}{\left(\lambda_{1}+x_{i1}^{\beta_{1}}\right)}^{-\gamma_{1}}-1\right)\left(2\lambda_{2}^{\gamma_{2}}{\left(\lambda_{2}+x_{i2}^{\beta_{2}}\right)}^{-\gamma_{2}}-1\right)\right]}^{2}}\} \end{array} \end{array}$$

$$\begin{array}{c} \begin{array}{cc} I_{\gamma_{l}\gamma_{j}}= & \sum_{i=1}^{n}\{4\theta \lambda_{l}^{\gamma_{l}}\lambda_{j}^{\gamma_{j}}{\left(\lambda_{l}+x_{il}^{\beta_{l}}\right)}^{-\gamma_{l}}{\left(\lambda_{j}+x_{ij}^{\beta_{j}}\right)}^{-\gamma_{j}}\\ & \;\frac{\left(\;\text{ln}\;\lambda_{l}-\;\text{ln}\;\left(\lambda_{l}+x_{il}^{\beta_{l}}\right)\right)\left(\;\text{ln}\;\lambda_{j}-\;\text{ln}\;\left(\lambda_{j}+x_{ij}^{\beta_{j}}\right)\right)}{1+\theta \left(2\lambda_{1}^{\gamma_{1}}{\left(\lambda_{1}+x_{i1}^{\beta_{1}}\right)}^{-\gamma_{1}}-1\right)\left(2\lambda_{2}^{\gamma_{2}}{\left(\lambda_{2}+x_{i2}^{\beta_{2}}\right)}^{-\gamma_{2}}-1\right)}\\ & \left[1-\frac{\theta \left(2\lambda_{l}^{\gamma_{l}}{\left(\lambda_{l}+x_{il}^{\beta_{l}}\right)}^{-\gamma_{l}}-1\right)\left(2\lambda_{j}^{\gamma_{j}}{\left(\lambda_{j}+x_{ij}^{\beta_{j}}\right)}^{-\gamma_{j}}-1\right)}{1+\theta \left(2\lambda_{1}^{\gamma_{1}}{\left(\lambda_{1}+x_{i1}^{\beta_{1}}\right)}^{-\gamma_{1}}-1\right)\left(2\lambda_{2}^{\gamma_{2}}{\left(\lambda_{2}+x_{i2}^{\beta_{2}}\right)}^{-\gamma_{2}}-1\right)}\right]\}, \end{array} \end{array}$$

$$\begin{array}{c} \begin{array}{cccc} I_{\gamma_{l}\beta_{l}}= & -\sum_{i=1}^{n}\frac{x_{il}^{\beta_{l}}\;\text{ln}\;\left(x_{il}\right)}{\left(\lambda_{l}+x_{il}^{\beta_{l}}\right)}-\sum_{i=1}^{n}\{2\theta \lambda_{l}^{\gamma_{l}}x_{il}\;\text{ln}\;\left(x_{il}\right){\left(\lambda_{l}+x_{il}^{\beta_{l}}\right)}^{-\gamma_{l}-1}\\ & \;\;\frac{\left(2\lambda_{j}^{\gamma_{j}}{\left(\lambda_{j}+x_{ij}^{\beta_{j}}\right)}^{-\gamma_{j}}-1\right)\left(\gamma_{l}\left(\;\text{ln}\;\lambda_{l}-\;\text{ln}\;\left(\lambda_{l}+x_{il}^{\beta_{l}}\right)\right)+1\right)}{1+\theta \left(2\lambda_{1}^{\gamma_{1}}{\left(\lambda_{1}+x_{i1}^{\beta_{1}}\right)}^{-\gamma_{1}}-1\right)\left(2\lambda_{2}^{\gamma_{2}}{\left(\lambda_{2}+x_{i2}^{\beta_{2}}\right)}^{-\gamma_{2}}-1\right)}\} & & \\ & +\sum_{i=1}^{n}\{4\theta^{2}\gamma_{l}\lambda_{l}^{2\gamma_{l}}x_{il}^{\beta_{l}}\;\text{ln}\;\left(x_{il}\right){\left(\lambda_{l}+x_{il}^{\beta_{l}}\right)}^{-2\gamma_{l}-1}\\ & \;\;\frac{{\left(2\lambda_{j}^{\gamma_{j}}{\left(\lambda_{j}+x_{ij}^{\beta_{j}}\right)}^{-\gamma_{j}}-1\right)}^{2}\left(\;\text{ln}\;\lambda_{l}-\;\text{ln}\;\left(\lambda_{l}+x_{il}^{\beta_{l}}\right)\right)}{{\left[1+\theta \left(2\lambda_{1}^{\gamma_{1}}{\left(\lambda_{1}+x_{i1}^{\beta_{1}}\right)}^{-\gamma_{1}}-1\right)\left(2\lambda_{2}^{\gamma_{2}}{\left(\lambda_{2}+x_{i2}^{\beta_{2}}\right)}^{-\gamma_{2}}-1\right)\right]}^{2}}\}, \end{array} \end{array}$$

$$\begin{array}{c} \begin{array}{cccc} I_{\gamma_{l}\lambda_{l}}= & \frac{n}{\lambda_{l}}-\sum_{i=1}^{n}\frac{1}{\left(\lambda_{l}+x_{il}^{\beta_{l}}\right)}+\sum_{i=1}^{n}\{\left(2\lambda_{j}^{\gamma_{j}}{\left(\lambda_{j}+x_{ij}^{\beta_{j}}\right)}^{-\gamma_{j}}-1\right){\left(\lambda_{l}+x_{il}^{\beta_{l}}\right)}^{-\gamma_{l}}\\ & \frac{2\theta \lambda_{l}^{\gamma_{l}}\left[\left(\lambda_{l}\left(\;\text{ln}\;\lambda_{l}-\;\text{ln}\;\left(\lambda_{l}+x_{il}^{\beta_{l}}\right)\right)+1\right)\left(\frac{1}{\lambda_{l}}-\frac{1}{\left(\lambda_{l}+x_{il}^{\beta_{l}}\right)}\right)\right]}{1+\theta \left(2\lambda_{1}^{\gamma_{1}}{\left(\lambda_{1}+x_{i1}^{\beta_{1}}\right)}^{-\gamma_{1}}-1\right)\left(2\lambda_{2}^{\gamma_{2}}{\left(\lambda_{2}+x_{i2}^{\beta_{2}}\right)}^{-\gamma_{2}}-1\right)}\} & & \\ & -\sum_{i=1}^{n}\{4\theta^{2}\gamma_{l}\lambda_{l}^{2\gamma_{l}}{\left(\lambda_{l}+x_{il}^{\beta_{l}}\right)}^{-2\gamma_{l}}{\left(2\lambda_{j}^{\gamma_{j}}{\left(\lambda_{j}+x_{ij}^{\beta_{j}}\right)}^{-\gamma_{j}}-1\right)}^{2}\\ & \frac{\left(\;\text{ln}\;\lambda_{l}-\;\text{ln}\;\left(\lambda_{l}+x_{il}^{\beta_{l}}\right)\right)\left(\frac{1}{\lambda_{l}}-\frac{1}{\left(\lambda_{l}+x_{il}^{\beta_{l}}\right)}\right)}{{\left[1+\theta \left(2\lambda_{1}^{\gamma_{1}}{\left(\lambda_{1}+x_{i1}^{\beta_{1}}\right)}^{-\gamma_{1}}-1\right)\left(2\lambda_{2}^{\gamma_{2}}{\left(\lambda_{2}+x_{i2}^{\beta_{2}}\right)}^{-\gamma_{2}}-1\right)\right]}^{2}}\}, \end{array} \end{array}$$

$$\begin{array}{c} \begin{array}{cccc} I_{\gamma_{l}\theta }= & \sum_{i=1}^{n}\frac{2\lambda_{l}^{\gamma_{l}}{\left(\lambda_{l}+x_{il}^{\beta_{l}}\right)}^{-\gamma_{l}}\left(2\lambda_{j}^{\gamma_{j}}{\left(\lambda_{j}+x_{ij}^{\beta_{j}}\right)}^{-\gamma_{j}}-1\right)\left(\;\text{ln}\;\lambda_{l}-\;\text{ln}\;\left(\lambda_{l}+x_{il}^{\beta_{l}}\right)\right)}{1+\theta \left(2\lambda_{1}^{\gamma_{1}}{\left(\lambda_{1}+x_{i1}^{\beta_{1}}\right)}^{-\gamma_{1}}-1\right)\left(2\lambda_{2}^{\gamma_{2}}{\left(\lambda_{2}+x_{i2}^{\beta_{2}}\right)}^{-\gamma_{2}}-1\right)} & & \\ & -\sum_{i=1}^{n}\{2\lambda_{l}^{\gamma_{l}}{\left(\lambda_{l}+x_{il}^{\beta_{l}}\right)}^{-\gamma_{l}}\left(2\lambda_{l}^{\gamma_{l}}{\left(\lambda_{l}+x_{il}^{\beta_{l}}\right)}^{-\gamma_{l}}-1\right)\\ & \frac{{\left(2\lambda_{j}^{\gamma_{j}}{\left(\lambda_{j}+x_{ij}^{\beta_{j}}\right)}^{-\gamma_{j}}-1\right)}^{2}\left(\;\text{ln}\;\lambda_{l}-\;\text{ln}\;\left(\lambda_{l}+x_{il}^{\beta_{l}}\right)\right)}{{\left[1+\theta \left(2\lambda_{1}^{\gamma_{1}}{\left(\lambda_{1}+x_{i1}^{\beta_{1}}\right)}^{-\gamma_{1}}-1\right)\left(2\lambda_{2}^{\gamma_{2}}{\left(\lambda_{2}+x_{i2}^{\beta_{2}}\right)}^{-\gamma_{2}}-1\right)\right]}^{2}}\}, \end{array} \end{array}$$

$$\begin{array}{c} \begin{array}{cccc} I_{\beta_{l}\beta_{l}}= & -\frac{n}{\beta_{l}^{2}}-\sum_{i=1}^{n}\frac{\left(\gamma_{l}+1\right)x_{il}^{\beta_{l}}{\left(\;\text{ln}\;x_{il}\right)}^{2}}{\lambda_{l}+x_{il}^{\beta_{l}}}-\sum_{i=1}^{n}\{\theta \gamma_{l}\lambda_{l}^{\gamma_{l}}x_{il}^{\beta_{l}}{\left(\;\text{ln}\;x_{il}\right)}^{2}\\ & \frac{{\left(\lambda_{l}+x_{il}^{\beta_{l}}\right)}^{-\gamma_{l}-1}\left(2\lambda_{j}^{\gamma_{j}}{\left(\lambda_{j}+x_{ij}^{\beta_{j}}\right)}^{-\gamma_{j}}-1\right)\left(1-\frac{\gamma_{l}+1}{\lambda_{l}+x_{il}^{\beta_{l}}}\right)}{1+\theta \left(2\lambda_{1}^{\gamma_{1}}{\left(\lambda_{1}+x_{i1}^{\beta_{1}}\right)}^{-\gamma_{1}}-1\right)\left(2\lambda_{2}^{\gamma_{2}}{\left(\lambda_{2}+x_{i2}^{\beta_{2}}\right)}^{-\gamma_{2}}-1\right)}\} & & \\ & -\sum_{i=1}^{n}\frac{{\left[2\theta \gamma_{l}\lambda_{l}^{\gamma_{l}}x_{il}^{\beta_{l}}\;\text{ln}\;\left(x_{il}\right){\left(\lambda_{l}+x_{il}^{\beta_{l}}\right)}^{-\gamma_{l}-1}\left(2\lambda_{j}^{\gamma_{j}}{\left(\lambda_{j}+x_{ij}^{\beta_{j}}\right)}^{-\gamma_{j}}-1\right)\right]}^{2}}{{\left[1+\theta \left(2\lambda_{1}^{\gamma_{1}}{\left(\lambda_{1}+x_{i1}^{\beta_{1}}\right)}^{-\gamma_{1}}-1\right)\left(2\lambda_{2}^{\gamma_{2}}{\left(\lambda_{2}+x_{i2}^{\beta_{2}}\right)}^{-\gamma_{2}}-1\right)\right]}^{2}} \end{array} \end{array}$$

$$\begin{array}{c} \begin{array}{cccc} I_{\beta_{l}\beta_{j}}= & \sum_{i=1}^{n}\frac{4\theta \gamma_{l}\gamma_{j}\lambda_{l}^{\gamma_{l}}\lambda_{j}^{\gamma_{j}}x_{il}^{\beta_{l}}x_{ij}^{\beta_{j}}\;\text{ln}\;\left(x_{il}\right)\;\text{ln}\;\left(x_{ij}\right){\left(\lambda_{l}+x_{il}^{\beta_{l}}\right)}^{-\gamma_{l}-1}{\left(\lambda_{j}+x_{ij}^{\beta_{j}}\right)}^{-\gamma_{j}-1}}{1+\theta \left(2\lambda_{1}^{\gamma_{1}}{\left(\lambda_{1}+x_{i1}^{\beta_{1}}\right)}^{-\gamma_{1}}-1\right)\left(2\lambda_{2}^{\gamma_{2}}{\left(\lambda_{2}+x_{i2}^{\beta_{2}}\right)}^{-\gamma_{2}}-1\right)} & & \\ & \left[1-\frac{\theta \left(2\lambda_{l}^{\gamma_{l}}{\left(\lambda_{l}+x_{il}^{\beta_{l}}\right)}^{-\gamma_{l}}-1\right)\left(2\lambda_{j}^{\gamma_{j}}{\left(\lambda_{j}+x_{ij}^{\beta_{j}}\right)}^{-\gamma_{j}}-1\right)}{1+\theta \left(2\lambda_{1}^{\gamma_{1}}{\left(\lambda_{1}+x_{i1}^{\beta_{1}}\right)}^{-\gamma_{1}}-1\right)\left(2\lambda_{2}^{\gamma_{2}}{\left(\lambda_{2}+x_{i2}^{\beta_{2}}\right)}^{-\gamma_{2}}-1\right)}\right], & \end{array} \end{array}$$

$$\begin{array}{c} \begin{array}{cccc} I_{\beta_{l}\lambda_{l}}= & \frac{\left(\lambda_{l}+1\right)x_{il}^{\beta_{l}}\;\text{ln}\;\left(x_{il}\right)}{{\left(\lambda_{l}+x_{il}^{\beta_{l}}\right)}^{2}}-\sum_{i=1}^{n}\{2\theta \gamma_{l}\lambda_{l}^{\gamma_{l}}x_{il}^{\beta_{l}}\;\text{ln}\;\left(x_{il}\right){\left(\lambda_{l}+x_{il}^{\beta_{l}}\right)}^{-\gamma_{l}-1}\\ & \frac{\left(2\lambda_{j}^{\gamma_{j}}{\left(\lambda_{j}+x_{ij}^{\beta_{j}}\right)}^{-\gamma_{j}}-1\right)\left(\frac{\gamma_{l}}{\lambda_{l}}-\frac{\left(\gamma_{l}+1\right)}{\left(\lambda_{l}+x_{il}^{\beta_{l}}\right)}\right)}{1+\theta \left(2\lambda_{1}^{\gamma_{1}}{\left(\lambda_{1}+x_{i1}^{\beta_{1}}\right)}^{-\gamma_{1}}-1\right)\left(2\lambda_{2}^{\gamma_{2}}{\left(\lambda_{2}+x_{i2}^{\beta_{2}}\right)}^{-\gamma_{2}}-1\right)}\} & & \\ & +\sum_{i=1}^{n}\{4\theta^{2}\gamma_{l}^{2}\lambda_{l}^{2\gamma_{l}}x_{il}^{\beta_{l}}\;\text{ln}\;\left(x_{il}\right){\left(\lambda_{l}+x_{il}^{\beta_{l}}\right)}^{-2\gamma_{l}-1}\\ & \frac{{\left(2\lambda_{j}^{\gamma_{j}}{\left(\lambda_{j}+x_{ij}^{\beta_{j}}\right)}^{-\gamma_{j}}-1\right)}^{2}\left(\frac{\gamma_{l}}{\lambda_{l}}-\frac{1}{\left(\lambda_{l}+x_{il}^{\beta_{l}}\right)}\right)}{{\left[1+\theta \left(2\lambda_{1}^{\gamma_{1}}{\left(\lambda_{1}+x_{i1}^{\beta_{1}}\right)}^{-\gamma_{1}}-1\right)\left(2\lambda_{2}^{\gamma_{2}}{\left(\lambda_{2}+x_{i2}^{\beta_{2}}\right)}^{-\gamma_{2}}-1\right)\right]}^{2}}\}, \end{array} \end{array}$$

$$\begin{array}{c} \begin{array}{cccc} I_{\beta_{l}\theta }=- & \sum_{i=1}^{n}\frac{2\gamma_{l}\lambda_{l}^{\gamma_{l}}x_{il}^{\beta_{l}}\;\text{ln}\;\left(x_{il}\right){\left(\lambda_{l}+x_{il}^{\beta_{l}}\right)}^{-\gamma_{l}-1}\left(2\lambda_{j}^{\gamma_{j}}{\left(\lambda_{j}+x_{ij}^{\beta_{j}}\right)}^{-\gamma_{j}}-1\right)}{1+\theta \left(2\lambda_{1}^{\gamma_{1}}{\left(\lambda_{1}+x_{i1}^{\beta_{1}}\right)}^{-\gamma_{1}}-1\right)\left(2\lambda_{2}^{\gamma_{2}}{\left(\lambda_{2}+x_{i2}^{\beta_{2}}\right)}^{-\gamma_{2}}-1\right)} & & \\ + & \sum_{i=1}^{n}\{2\theta \gamma_{l}\lambda_{l}^{\gamma_{l}}x_{il}^{\beta_{l}}\;\text{ln}\;\left(x_{il}\right){\left(\lambda_{l}+x_{il}^{\beta_{l}}\right)}^{-\gamma_{l}-1}\\ & \frac{\left(2\lambda_{l}^{\gamma_{l}}{\left(\lambda_{l}+x_{il}^{\beta_{l}}\right)}^{-\gamma_{l}}-1\right){\left(2\lambda_{j}^{\gamma_{j}}{\left(\lambda_{j}+x_{ij}^{\beta_{j}}\right)}^{-\gamma_{j}}-1\right)}^{2}}{{\left[1+\theta \left(2\lambda_{1}^{\gamma_{1}}{\left(\lambda_{1}+x_{i1}^{\beta_{1}}\right)}^{-\gamma_{1}}-1\right)\left(2\lambda_{2}^{\gamma_{2}}{\left(\lambda_{2}+x_{i2}^{\beta_{2}}\right)}^{-\gamma_{2}}-1\right)\right]}^{2}}\}, \end{array} \end{array}$$

$$\begin{array}{c} \begin{array}{cccc} I_{\lambda_{l}\lambda_{l}}= & -\frac{n\gamma_{l}}{\lambda_{l}^{2}}+\sum_{i=1}^{n}\frac{\gamma_{l}+1}{{\left(\lambda_{l}+x_{il}^{\beta_{l}}\right)}^{2}}+\sum_{i=1}^{n}\{2\theta \gamma_{l}\lambda_{l}^{\gamma_{l}}{\left(\lambda_{l}+x_{il}^{\beta_{l}}\right)}^{-\gamma_{l}}\\ & \frac{\left(2\lambda_{j}^{\gamma_{j}}{\left(\lambda_{j}+x_{ij}^{\beta_{j}}\right)}^{-\gamma_{j}}-1\right)\left(\frac{\gamma_{l}-1}{\lambda_{l}}-\frac{\gamma_{l}+1}{\left(\lambda_{l}+x_{il}^{\beta_{l}}\right)}\right)\left(\frac{1}{\lambda_{l}}-\frac{1}{\left(\lambda_{l}+x_{il}^{\beta_{l}}\right)}\right)}{1+\theta \left(2\lambda_{1}^{\gamma_{1}}{\left(\lambda_{1}+x_{i1}^{\beta_{1}}\right)}^{-\gamma_{1}}-1\right)\left(2\lambda_{2}^{\gamma_{2}}{\left(\lambda_{2}+x_{i2}^{\beta_{2}}\right)}^{-\gamma_{2}}-1\right)}\} & & \\ & -\sum_{i=1}^{n}\{4\theta^{2}\gamma_{l}^{2}\lambda_{l}^{2\gamma_{l}}{\left(\lambda_{l}+x_{il}^{\beta_{l}}\right)}^{-2\gamma_{l}}\\ & \frac{{\left(2\lambda_{j}^{\gamma_{j}}{\left(\lambda_{j}+x_{ij}^{\beta_{j}}\right)}^{-\gamma_{j}}-1\right)}^{2}{\left(\frac{1}{\lambda_{l}}-\frac{1}{\left(\lambda_{l}+x_{il}^{\beta_{l}}\right)}\right)}^{2}}{{\left[1+\theta \left(2\lambda_{1}^{\gamma_{1}}{\left(\lambda_{1}+x_{i1}^{\beta_{1}}\right)}^{-\gamma_{1}}-1\right)\left(2\lambda_{2}^{\gamma_{2}}{\left(\lambda_{2}+x_{i2}^{\beta_{2}}\right)}^{-\gamma_{2}}-1\right)\right]}^{2}}\}, \end{array} \end{array}$$

$$\begin{array}{c} \begin{array}{cccc} I_{\lambda_{l}\lambda_{j}}= & -\frac{n\gamma_{l}}{\lambda_{l}^{2}}+\sum_{i=1}^{n}\frac{\gamma_{l}+1}{{\left(\lambda_{l}+x_{il}^{\beta_{l}}\right)}^{2}}+\sum_{i=1}^{n}\{2\theta \gamma_{l}\lambda_{l}^{\gamma_{l}}{\left(\lambda_{l}+x_{il}^{\beta_{l}}\right)}^{-\gamma_{l}}\\ & \frac{\left(2\lambda_{j}^{\gamma_{j}}{\left(\lambda_{j}+x_{ij}^{\beta_{j}}\right)}^{-\gamma_{j}}-1\right)\left(\frac{1}{\lambda_{l}}-\frac{1}{\left(\lambda_{l}+x_{il}^{\beta_{l}}\right)}\right)\left(\frac{\gamma_{l}-1}{\lambda_{l}}-\frac{\gamma_{l}+1}{\left(\lambda_{l}+x_{il}^{\beta_{l}}\right)}\right)}{1+\theta \left(2\lambda_{1}^{\gamma_{1}}{\left(\lambda_{1}+x_{i1}^{\beta_{1}}\right)}^{-\gamma_{1}}-1\right)\left(2\lambda_{2}^{\gamma_{2}}{\left(\lambda_{2}+x_{i2}^{\beta_{2}}\right)}^{-\gamma_{2}}-1\right)}\} & & \\ & -\sum_{i=1}^{n}\{4\theta^{2}\gamma_{l}^{2}\lambda_{l}^{2\gamma_{l}}{\left(\lambda_{l}+x_{il}^{\beta_{l}}\right)}^{-2\gamma_{l}}\\ & \frac{{\left(2\lambda_{j}^{\gamma_{j}}{\left(\lambda_{j}+x_{ij}^{\beta_{j}}\right)}^{-\gamma_{j}}-1\right)}^{2}{\left(\frac{1}{\lambda_{l}}-\frac{1}{\left(\lambda_{l}+x_{il}^{\beta_{l}}\right)}\right)}^{2}}{{\left[1+\theta \left(2\lambda_{1}^{\gamma_{1}}{\left(\lambda_{1}+x_{i1}^{\beta_{1}}\right)}^{-\gamma_{1}}-1\right)\left(2\lambda_{2}^{\gamma_{2}}{\left(\lambda_{2}+x_{i2}^{\beta_{2}}\right)}^{-\gamma_{2}}-1\right)\right]}^{2}}\}, \end{array} \end{array}$$

$$\begin{array}{c} \begin{array}{cccc} I_{\lambda_{l}\theta }= & \sum_{i=1}^{n}\frac{2\gamma_{l}\lambda_{l}^{\gamma_{l}}{\left(\lambda_{l}+x_{il}^{\beta_{l}}\right)}^{-\gamma_{l}}\left(2\lambda_{j}^{\gamma_{j}}{\left(\lambda_{j}+x_{ij}^{\beta_{j}}\right)}^{-\gamma_{j}}-1\right)\left(\frac{1}{\lambda_{l}}-\frac{1}{\left(\lambda_{l}+x_{il}^{\beta_{l}}\right)}\right)}{1+\theta \left(2\lambda_{1}^{\gamma_{1}}{\left(\lambda_{1}+x_{i1}^{\beta_{1}}\right)}^{-\gamma_{1}}-1\right)\left(2\lambda_{2}^{\gamma_{2}}{\left(\lambda_{2}+x_{i2}^{\beta_{2}}\right)}^{-\gamma_{2}}-1\right)} & & \\ & -\sum_{i=1}^{n}\{2\gamma_{l}\lambda_{l}^{\gamma_{l}}{\left(\lambda_{l}+x_{il}^{\beta_{l}}\right)}^{-\gamma_{l}}{\left(2\lambda_{j}^{\gamma_{j}}{\left(\lambda_{j}+x_{ij}^{\beta_{j}}\right)}^{-\gamma_{j}}-1\right)}^{2}\\ & \frac{\left(2\lambda_{l}^{\gamma_{l}}{\left(\lambda_{l}+x_{il}^{\beta_{l}}\right)}^{-\gamma_{l}}-1\right)\left(\frac{1}{\lambda_{l}}-\frac{1}{\left(\lambda_{l}+x_{il}^{\beta_{l}}\right)}\right)}{{\left[1+\theta \left(2\lambda_{1}^{\gamma_{1}}{\left(\lambda_{1}+x_{i1}^{\beta_{1}}\right)}^{-\gamma_{1}}-1\right)\left(2\lambda_{2}^{\gamma_{2}}{\left(\lambda_{2}+x_{i2}^{\beta_{2}}\right)}^{-\gamma_{2}}-1\right)\right]}^{2}}\}, \end{array} \end{array}$$

$$\begin{array}{c} \begin{array}{ccc} I_{\theta \theta }= & -\sum_{i=1}^{n}\frac{{\left(2\lambda_{1}^{\gamma_{1}}{\left(\lambda_{1}+x_{i1}^{\beta_{1}}\right)}^{-\gamma_{1}}-1\right)}^{2}{\left(2\lambda_{2}^{\gamma_{2}}{\left(\lambda_{2}+x_{i2}^{\beta_{2}}\right)}^{-\gamma_{2}}-1\right)}^{2}}{{\left[1+\theta \left(2\lambda_{1}^{\gamma_{1}}{\left(\lambda_{1}+x_{i1}^{\beta_{1}}\right)}^{-\gamma_{1}}-1\right)\left(2\lambda_{2}^{\gamma_{2}}{\left(\lambda_{2}+x_{i2}^{\beta_{2}}\right)}^{-\gamma_{2}}-1\right)\right]}^{2}}. & \end{array} \end{array}$$

### 7.2 Highest posterior density Bayesian estimation

The approach of Chen and Shao [39] is frequently used to construct highest posterior density (HPD) intervals for unknown distribution parameters in Bayesian estimation. For instance, two endpoints from the MCMC sampling outputs, the lower 5% and upper 95% percentiles, can be used to calculate a 90% HPD interval. The following is how reliable Bayesian intervals of the parameters are obtained:

1. Arrange Θ_*j*_; *j* = 1, 2, …, 7 as $\gamma_{l}^{\left[1\right]}<\gamma_{l}^{\left[2\right]}<\ldots <\gamma_{l}^{\left[L\right]},\beta_{l}^{\left[1\right]}<\beta_{l}^{\left[2\right]}<\ldots <\beta_{l}^{\left[L\right]},\lambda_{l}^{\left[1\right]}<\lambda_{l}^{\left[2\right]}<\ldots <\lambda_{l}^{\left[L\right]}$ and *θ*^[1]^ < *θ*^[2]^ < … < *θ*^[*L*]^ where *l* = 1, 2, 3, and *L* is the length of MCMC generated.
2. The 90% symmetric reliable intervals of *γ*_1_, *β*_1_, λ_1_, *γ*_2_, *β*_2_, λ_2_ and *θ* become $\left(\gamma_{l}^{L_{500/10,000}},\gamma_{l}^{L_{9500/10,000}}\right),\left(\beta_{l}^{L_{500/10,000}},\beta_{l}^{L_{9500/10,000}}\right),\left(\lambda_{l}^{L_{500/10,000}},\lambda_{l}^{L_{9500/10,000}}\right)$ and $\left(\theta^{L_{500/10,000}},\theta^{L_{9500/10,000}}\right)$.

## 8 Simulation

The performance of MLE using the Newton—Raphson (NR) and Bayesian estimates using the Metropolis—Hastings methods are contrasted numerically in this section. For the parameters of the BFGMPLx distribution, the performance of the various techniques and the analytically deduced results may be evaluated exactly. The “maxLik” package was installed in R 4.2.2 programming, and after 1000 samples from BFGMPLx distribution had been gathered, the MLEs and 95% ACI estimations of the parameter values were assessed. To acquire the Bayes point estimates and their HPD interval estimates of the same unknown parameters using the “coda” tool in the R programming language. For simulation study of BFGMPLx distribution, the values of the parameter can be defined as follows:

In Table 1: *γ*_1_ = 0.6;*β*_1_ = 3, λ_1_ = 2.8, *γ*_2_ = 0.9, *β*_2_ = 2.5, λ_2_ = 0.7.

In Table 2: *γ*_1_ = 1.3, *β*_1_ = 5, λ_1_ = 1.5, *γ*_2_ = 0.9, *β*_2_ = 4, λ_2_ = 1.3.

In Table 3: *γ*_1_ = 0.8;*β*_1_ = 1.3, λ_1_ = 1.5, *γ*_2_ = 1.2, *β*_2_ = 1.5, λ_2_ = 2.

While *θ* is a copula parameter is changed in all tables as 0.4, and -0.5. The sample-sizes (n) are as follows 35, 50, 100, and 150. The simulation results of bias, mean squared error (MSE), and length of CI (LCI) (For MLE asymptotic CI (LACI), while Bayesian credible CI (LCCI)) on 5000 iteration of Monte Carlo simulation are shown in Tables 1–3. Nelsen [8] has explored the construction of a sample from a defined joint distribution and utilising a conditional technique to generate random variables. The generated samples are used to calculate the Bayesian estimates, together with the corresponding posterior symmetric loss function and Bayesian HPD intervals.

**Table 1:** MLE and Bayesian estimation method for BFGMPLx model parameters.

*γ*_1_ = 0.6;*β*_1_ = 3, λ_1_ = 2.8, *γ*_2_ = 0.9, *β*_2_ = 2.5, λ_2_ = 0.7													
*θ*		0.4						-0.5					
		MLE			Bayesian			MLE			Bayesian		
n		Bias	MSE	LACI	Bias	MSE	LCCI	Bias	MSE	LACI	Bias	MSE	LCCI
35	*γ*_1_	0.0426	0.1388	1.4513	0.0691	0.0478	0.7020	0.0725	0.1676	1.5803	0.0822	0.0516	0.7745
	*β*_1_	0.5780	1.4556	4.1534	0.2914	0.4496	2.0214	0.5442	1.2851	3.9003	0.3002	0.4732	2.1138
	λ_1_	1.2769	6.4416	6.2356	1.1300	5.3835	5.3019	1.3186	7.4335	6.8793	1.1745	4.4663	6.0414
	*γ*_2_	0.2016	0.7905	3.3962	0.1387	0.1256	1.1547	0.1519	0.6745	3.1654	0.0989	0.0897	1.0232
	*β*_2_	0.5943	1.4096	4.0310	0.2425	0.3439	1.8507	0.6618	1.5534	4.1420	0.2844	0.3609	1.8969
	λ_2_	0.3574	1.3276	1.4513	0.2818	0.2857	0.7020	0.3355	1.4495	1.5803	0.2472	0.2415	0.7745
	*θ*	-0.0039	0.6426	4.1534	-0.0471	0.1708	2.0214	0.0375	0.4511	3.9003	0.0754	0.2074	2.1138
50	*γ*_1_	0.0351	0.1001	1.2329	0.0537	0.0300	0.5669	0.0260	0.0944	1.2009	0.0591	0.0319	0.6073
	*β*_1_	0.4141	0.8822	3.3064	0.1982	0.2134	1.5731	0.4782	1.0331	3.5177	0.2397	0.2723	1.6571
	λ_1_	0.9873	5.3741	6.2262	0.9839	3.3314	5.2976	0.7728	5.0360	6.2629	0.8845	2.9762	5.0097
	*γ*_2_	0.1220	0.3774	2.3612	0.0978	0.0798	0.9588	0.1355	0.4825	2.6718	0.1074	0.0902	0.9827
	*β*_2_	0.3855	0.7384	3.0122	0.1855	0.1892	1.4269	0.4377	0.8143	3.0950	0.1910	0.1920	1.5366
	λ_2_	0.2388	0.6755	1.2329	0.2197	0.2143	0.5669	0.2389	0.8523	1.2009	0.2055	0.1780	0.6073
	*θ*	-0.0472	0.2341	3.3064	-0.0623	0.1395	1.5731	0.0541	0.3028	3.5177	0.0851	0.1579	1.6571
100	*γ*_1_	0.0066	0.0373	0.7573	0.0359	0.0120	0.3617	0.0236	0.0496	0.8690	0.0502	0.0154	0.4046
	*β*_1_	0.2307	0.3879	2.2689	0.1024	0.0868	1.0571	0.2701	0.4546	2.4230	0.1200	0.0937	1.0540
	λ_1_	0.4065	2.0207	5.3423	0.6037	1.4423	3.6030	0.4731	2.5400	5.9688	0.6253	1.6565	3.7336
	*γ*_2_	0.0485	0.1593	1.5537	0.0727	0.0374	0.6419	0.0194	0.1471	1.5022	0.0721	0.0424	0.6621
	*β*_2_	0.1872	0.2630	1.8725	0.0628	0.0553	0.8571	0.2602	0.3149	1.9498	0.1019	0.0696	0.9012
	λ_2_	0.0988	0.2926	0.7573	0.1430	0.0922	0.3617	0.0677	0.2478	0.8690	0.1503	0.0982	0.4046
	*θ*	-0.0375	0.0981	2.2689	-0.0433	0.0794	1.0571	0.0612	0.1011	2.4230	0.0784	0.0953	1.0540
150	*γ*_1_	0.0105	0.0285	0.6612	0.0251	0.0062	0.2758	0.0274	0.0295	0.6646	0.0359	0.0076	0.3150
	*β*_1_	0.1516	0.2645	1.9276	0.0666	0.0522	0.8109	0.1254	0.2366	1.8432	0.0650	0.0517	0.8478
	λ_1_	0.2962	1.2453	4.2197	0.4179	0.7738	2.4479	0.3194	1.2420	4.1873	0.3992	0.7463	2.4802
	*γ*_2_	0.0416	0.0936	1.1889	0.0652	0.0273	0.5418	0.0046	0.0826	1.1267	0.0579	0.0261	0.5101
	*β*_2_	0.1123	0.1377	1.3872	0.0364	0.0305	0.6312	0.1885	0.1855	1.5187	0.0671	0.0378	0.6847
	λ_2_	0.0856	0.1742	0.6612	0.1275	0.0710	0.2758	0.0338	0.1441	0.6646	0.1193	0.0690	0.3150
	*θ*	-0.0326	0.0623	1.9276	-0.0390	0.0592	0.8109	0.0506	0.0587	1.8432	0.0478	0.0462	0.8478

**Table 2:** MLE and Bayesian estimation method for BFGMPLx model parameters.

*γ*_1_ = 1.3, *β*_1_ = 5, λ_1_ = 1.5, *γ*_2_ = 0.9, *β*_2_ = 4, λ_2_ = 1.3													
*θ*		0.4						-0.5					
		MLE			Bayesian			MLE			Bayesian		
n		Bias	MSE	LACI	Bias	MSE	LCCI	Bias	MSE	LACI	Bias	MSE	LCCI
35	*γ*_1_	0.5911	2.4492	5.6832	0.2666	0.3575	1.8784	0.4886	2.3494	5.6979	0.2394	0.3185	1.7747
	*β*_1_	0.3675	1.8207	5.0920	0.2022	1.5371	2.5418	0.5203	2.4058	5.7308	0.1725	0.4848	2.5303
	λ_1_	1.0346	7.0430	9.5848	0.5267	1.1434	3.3760	0.8877	6.7168	9.5497	0.4975	1.0268	3.0952
	*γ*_2_	0.4203	1.6466	4.7550	0.1484	0.1332	1.2152	0.4729	2.0135	5.2470	0.1519	0.1365	1.2032
	*β*_2_	0.3818	1.6999	4.8893	0.1431	0.3932	2.3152	0.3775	1.7347	4.9488	0.1560	0.4225	2.3267
	λ_2_	0.9728	7.3869	5.6832	0.4329	0.8226	1.8784	1.1088	9.6497	5.6979	0.4084	0.7180	1.7747
	*θ*	0.0413	0.4969	5.0920	-0.0113	0.1646	2.5418	-0.0965	0.5467	5.7308	0.0435	0.2058	2.5303
50	*γ*_1_	0.4258	1.5997	4.6709	0.2036	0.2537	1.7009	0.4803	1.8082	4.9259	0.2080	0.2384	1.5910
	*β*_1_	0.2988	1.3841	4.4628	0.1445	0.3187	2.0055	0.2663	1.3509	4.4371	0.1407	0.2966	1.9489
	λ_1_	0.7316	4.2554	7.5646	0.4159	0.8428	2.8538	0.8312	4.8908	8.0375	0.4286	0.8123	2.8644
	*γ*_2_	0.2815	0.9716	3.7048	0.1317	0.0989	1.0212	0.2849	0.9391	3.6326	0.1295	0.0998	0.9945
	*β*_2_	0.3143	1.2729	4.2497	0.1231	0.2543	1.8384	0.2415	1.0648	3.9346	0.0849	0.2493	1.8923
	λ_2_	0.6520	4.2589	4.6709	0.3810	0.6308	1.7009	0.6727	4.5123	4.9259	0.3548	0.5925	1.5910
	*θ*	0.0181	0.2206	4.4628	-0.0353	0.1291	2.0055	-0.0027	0.2174	4.4371	0.0828	0.1494	1.9489
100	*γ*_1_	0.1819	0.4765	2.6117	0.1666	0.1310	1.0806	0.2216	0.5830	2.8657	0.1551	0.1341	1.1960
	*β*_1_	0.1580	0.5993	2.9723	0.0361	0.1198	1.2791	0.1117	0.6543	3.1420	0.0214	0.1207	1.3614
	λ_1_	0.3441	1.4668	4.5542	0.3431	0.5016	2.0445	0.3772	1.5870	4.7141	0.2985	0.4244	1.9489
	*γ*_2_	0.1528	0.2648	1.9273	0.0935	0.0460	0.6864	0.1144	0.2439	1.8844	0.0925	0.0477	0.7142
	*β*_2_	0.0929	0.5119	2.7824	0.0275	0.0922	1.1363	0.1100	0.4956	2.7272	0.0086	0.0876	1.1230
	λ_2_	0.3592	1.3248	2.6117	0.2583	0.3030	1.0806	0.2760	1.1714	2.8657	0.2543	0.2968	1.1960
	*θ*	0.0066	0.0900	2.9723	-0.0251	0.0788	1.2791	-0.0078	0.0907	3.1420	0.0399	0.0868	1.3614
150	*γ*_1_	0.1344	0.3112	2.1233	0.1296	0.0898	0.9081	0.1664	0.3564	2.2485	0.1368	0.0947	0.9596
	*β*_1_	0.1021	0.4184	2.5050	0.0156	0.0870	1.1265	0.0605	0.3736	2.3855	-0.0024	0.0795	1.0800
	λ_1_	0.2231	0.8294	3.4631	0.2428	0.2794	1.6600	0.2821	0.9681	3.6969	0.2496	0.2933	1.7005
	*γ*_2_	0.0768	0.1082	1.2544	0.0692	0.0271	0.5292	0.0738	0.1274	1.3696	0.0693	0.0283	0.5154
	*β*_2_	0.0713	0.3077	2.1575	0.0150	0.0564	0.9336	0.0682	0.3096	2.1658	0.0001	0.0570	0.9172
	λ_2_	0.1836	0.5201	2.1233	0.1897	0.1652	0.9081	0.1773	0.5997	2.2485	0.1958	0.1901	0.9596
	*θ*	0.0090	0.0546	2.5050	-0.0041	0.0530	1.1265	-0.0111	0.0605	2.3855	0.0278	0.0578	1.0800

**Table 3:** MLE and Bayesian estimation method for BFGMPLx model parameters.

*γ*_1_ = 0.8;*β*_1_ = 1.3, λ_1_ = 1.5, *γ*_2_ = 1.2, *β*_2_ = 1.5, λ_2_ = 2													
*θ*		0.4						-0.5					
		MLE			Bayesian			MLE			Bayesian		
n		Bias	MSE	LACI	Bias	MSE	LCCI	Bias	MSE	LACI	Bias	MSE	LCCI
35	*γ*_1_	-0.5312	0.2905	0.3580	-0.0644	0.0080	0.2373	-0.4997	0.2615	0.4248	-0.0660	0.0115	0.2887
	*β*_1_	2.9669	9.5983	3.4991	0.7608	0.7168	1.4058	3.0384	10.0231	3.4887	0.9441	1.0761	1.6540
	λ_1_	1.7325	12.5103	12.0939	1.5496	4.1261	4.2565	1.8929	15.7237	13.6655	1.5036	4.1946	4.4399
	*γ*_2_	-0.5891	0.4668	1.3570	-0.0155	0.0590	0.8443	-0.6414	0.5092	1.2264	-0.0257	0.0456	0.7146
	*β*_2_	2.0352	5.6060	4.7455	0.7129	0.6954	1.6412	2.4550	7.7772	5.1886	0.8566	1.0058	1.9630
	λ_2_	0.3222	4.6438	0.3580	1.1521	3.2962	0.2373	0.2845	6.4787	0.4248	1.1819	3.5457	0.2887
	*θ*	-0.1525	0.4103	3.4991	-0.0574	0.1609	1.4058	0.3394	0.5607	3.4887	0.2282	0.2394	1.6540
50	*γ*_1_	-0.5430	0.2852	0.2612	-0.0697	0.0066	0.1367	-0.5143	0.2617	0.3141	-0.0722	0.0075	0.1725
	*β*_1_	3.0197	9.6453	2.8468	0.7577	0.6688	1.1741	3.0417	9.7998	2.9021	0.9195	0.9706	1.3539
	λ_1_	1.4949	8.5360	11.3000	1.5822	4.1078	4.1954	1.5225	11.4249	11.8357	1.4727	3.8437	4.1534
	*γ*_2_	-0.6197	0.4540	1.0380	-0.0428	0.0486	0.6167	-0.6447	0.4744	0.9504	-0.0523	0.0328	0.5387
	*β*_2_	1.9305	4.7485	3.9643	0.6788	0.5902	1.3416	2.3231	6.5405	4.1946	0.8603	0.9238	1.5691
	λ_2_	0.0326	2.6685	0.2612	1.0354	2.6236	0.1367	-0.0348	3.4386	0.3141	1.0000	2.5066	0.1725
	*θ*	-0.1905	0.2489	2.8468	-0.0793	0.1271	1.1741	0.4051	0.3469	2.9021	0.2533	0.2080	1.3539
100	*γ*_1_	-0.4550	0.2630	0.1669	-0.0736	0.0059	0.0792	-0.5226	0.2476	0.1986	-0.0806	0.0070	0.0883
	*β*_1_	1.0544	4.6300	2.1498	0.7757	0.6471	0.8038	3.1055	9.9033	1.9965	0.9758	0.9011	0.9313
	λ_1_	1.0902	4.8045	7.4579	1.7119	4.0272	3.9037	1.2054	6.0199	8.3814	1.6228	3.6989	3.8775
	*γ*_2_	-0.6433	0.4422	0.6598	-0.0880	0.0138	0.2699	-0.6969	0.4502	0.4937	-0.0987	0.0132	0.2420
	*β*_2_	1.8054	3.7853	2.8444	0.6674	0.5104	0.9709	2.3072	5.8966	2.9696	0.8746	0.8576	1.1597
	λ_2_	-0.3690	1.2164	0.1669	0.3176	1.0395	0.0792	-0.3962	1.5893	0.1986	0.8254	1.5949	0.0883
	*θ*	-0.2319	0.1370	2.1498	-0.1112	0.0826	0.8038	0.4044	0.2484	1.9965	0.2990	0.1667	0.9313
150	*γ*_1_	-0.3555	0.2309	0.1376	-0.0727	0.0056	0.0656	-0.5265	0.2379	0.1564	-0.0817	0.0070	0.0669
	*β*_1_	0.9075	1.6607	1.7731	0.7622	0.6140	0.6986	3.0951	9.7743	1.7313	0.9674	0.8973	0.7489
	λ_1_	0.9158	3.5744	6.4871	0.6748	3.1959	3.6681	0.9071	3.8062	6.7741	1.5379	3.4504	3.5993
	*γ*_2_	-0.5590	0.4347	0.5132	-0.1052	0.0114	0.2065	-0.7048	0.4451	0.3972	-0.1090	0.0131	0.1623
	*β*_2_	1.7656	3.4809	2.3648	0.6625	0.4854	0.8442	2.2586	5.4907	2.4469	0.8599	0.8033	0.9664
	λ_2_	-0.4857	0.7491	0.1376	0.3664	0.6881	0.0656	-0.5930	1.0122	0.1564	0.6653	0.9706	0.0669
	*θ*	-0.2272	0.1036	1.7731	-0.1141	0.0712	0.6986	0.4043	0.2165	1.7313	0.3245	0.1658	0.7489

The following conclusions can be drawn from Tables 1–3:

- It can be shown that MLE and Bayesian estimates of unknown parameters are fairly good in terms of Bias, MSE, and LCI.
- With an increase in sample size, it is shown that the posterior risks decrease and the estimated values of the parameters approach the nominal values of the parameters.
- Since Bayesian estimates incorporate prior knowledge based on gamma informative priors, they are superior to MLE in terms of bias, MSE, and LCI.
- Additionally, the HPD credible intervals beat the ACI in terms of LCI outcomes when the sample size n increases.

## 9 Application

The fit models were compared using the conventional value of criteria (VC), which included the Akaike information criterion (AIVC), consistent AIVC (CAIVC), Bayesian information criterion (BIVC), Hannan-Quinn information criterion (HQIVC), Anderson-Darling value (ADV), Cramer-von Mises value (CMV), Kolmogorov-Smirnov distance (KSD), p-value of Kolmogorov-S (PVKS), and standard error (SE). Our main statistical objective was to analyse a genuine dataset that is important in various domains using a fitting approach model. In this regard, we contrasted the suggested BFGMPLx distribution’s fit with the BFGMWE and BFGMWITL mentioned by El-Sherpieny et al. [24] and Bivariate FGM Lomax-Claim (BFGMLC) distribution which was mentioned by Zhao et al. [40].

We have thought about the length of diabetes and serum creatinine in this part (SrCr). Since the patients’ diabetes was already known, we are predicting the complications that may result from it (using the values of SrCr, the data has been divided into two groups: groups with diabetic nephropathy (DN) (SrCr 1.4mg/dl) and groups with nondiabetic nephropathy (SrCr 1.4mg/dl)). SrCr reports were provided for each patient from the 200 patients whose reports were available. From January 2012 to August 2013, the pathology reports of these patients were gathered from the path lab of Dr. Lal. This data has been discussed by Grover et al. [41]. Duration of diabetes: 7.4, 9, 10, 11, 12, 13, 13.75, 14.92, 15.8286, 16.9333, 18, 19, 20, 21, 22, 23, 24, 26, 26.6. Serum Creatinine: 1.925, 1.5, 2, 1.6, 1.7, 1.7533, 1.54, 1.694, 1.8843, 1.8433, 1.832, 1.59, 1.7833, 1.2, 1.792, 1.5, 1.5033, 2, 2.14.

**Fig 8:**
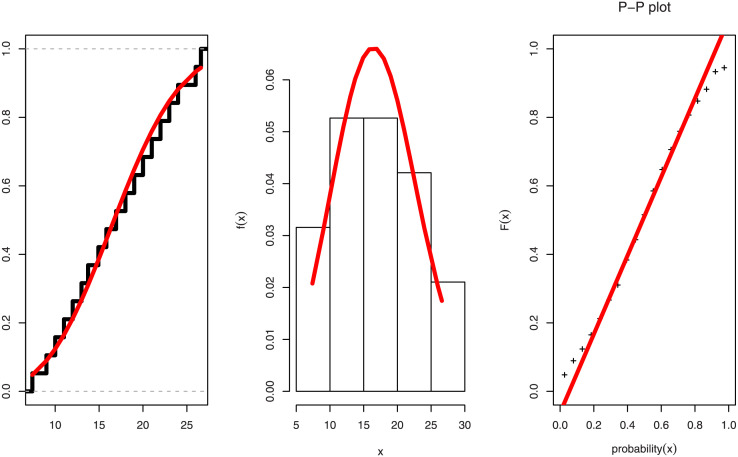
Estimated cdf, pdf and pp-plot for duration of diabetes data.

**Fig 9:**
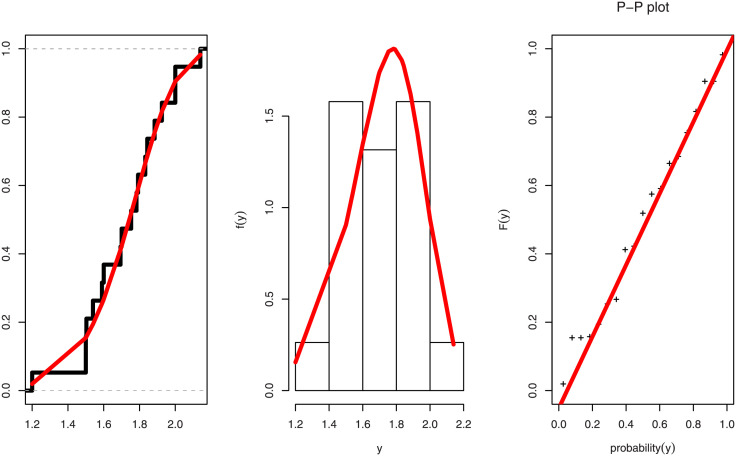
Estimated cdf, pdf and pp-plot for stress variable for serum creatinine data.

Table 4 lists the MLE, SE, KS distance and associated p-value, CVM and AD for the marginal distributions. The fitted pdf with histogram plot, fitted cdf with empirical cdf and P-P plot of PL distribution in Figs 8 and 9 for Duration of diabetes and Serum Creatinine respectively support the findings (KS-test) in Table 4 of our study.

**Table 4:** MLE with different measures of Goodness of fit test of marginal distribution.

		Estimates	SE	CVM	AD	KSD	PVKS
X	*γ*	11.3951	20.1213	0.0216	0.1659	0.0751	0.9996
	*β*	3.2833	0.6177				
	λ	164764.8045	2918.7836				
Y	*γ*	8.0453	33.3879	0.0248	0.1915	0.1020	0.9890
	*β*	9.6562	3.1980				
	λ	2377.1949	741.5255				

Figs 8 and 9 show the fitted CDF with empirical CDF, fitted PDF with histogram plot, and PP-plot for the duration of diabetes and serum creatinine, respectively. In Table 5 goodness of fit measures for FGM copula have been obtained as kendall, sperman, tau, rho, statistics, and copula parameter *θ* with P-Value is 0.1339 > 0.05, then we accept the null hypothesises as the bivariate date is fit for FGM copula. The MLE with SE, and coefficient of variation (CV) for parameters of BFGMPLx, BFGMWE, BFGMWITL, and BFGMLC distributions have been discussed in Table 6. Table 7 obtained AIC, BIC, HQIC, and CAIC for bivariate models to select the best fit bivariate model of this data.

**Table 5:** Goodness of fit measures for FGM copula.

	kendall	spearman	tau	rho	statistic	parameter	p-value
FGM	0.0176	0.0290	0.0146	0.0220	0.4807	0.0659	0.1339

**Table 6:** MLE, SE, and CV for parameters of bivariate distributions.

		*γ*_1_	*β*_1_	λ_1_	*γ*_2_	*β*_2_	λ_2_	*θ*
BFGMPLx	estimate	11.1515	3.2939	166619.0615	7.9844	9.6625	2369.9352	0.0438
	SE	9.4498	0.6113	2915.7202	3.5112	3.2020	556.4657	0.0507
	CV	84.74%	18.56%	1.75%	43.98%	33.14%	23.48%	115.65%
BFGMWE	estimate	133.8298	3.1032	0.0098	0.0290	5.5988	0.5796	0.0750
	SE	5.8496	0.4250	0.0019	0.1381	1.9469	0.3695	0.0957
	CV	4.37%	13.69%	19.81%	476.32%	34.77%	63.75%	127.60%
BFGMWITL	estimate	0.0157	4.8459	0.5208	5.5389	7.2635	1.0806	0.0528
	SE	0.0093	1.3815	0.1874	2.2875	1.4924	0.4932	0.0558
	CV	59.34%	28.51%	35.97%	41.30%	20.55%	45.65%	105.56%
BFGMLC	estimate	25.2494	0.0118	0.0116	36.7275	0.3049	0.0002	0.0694
	SE	19.2562	0.0107	0.1152	4.1459	0.2052	0.0003	0.0518
	CV	76.26%	90.48%	994.49%	11.29%	67.28%	188.17%	74.70%

**Table 7:** AIC, BIC, HQIC, and CAIC for bivariate models.

	AIC	BIC	HQIC	CAIC
BFGMPLx	128.7305	137.2087	134.8693	129.7694
BFGMWE	129.6834	139.8652	136.2945	130.8022
BFGMWITL	129.3130	139.4948	135.9240	130.4318
BFGMLC	132.6032	142.7851	139.2143	133.7221

**Fig 10:**
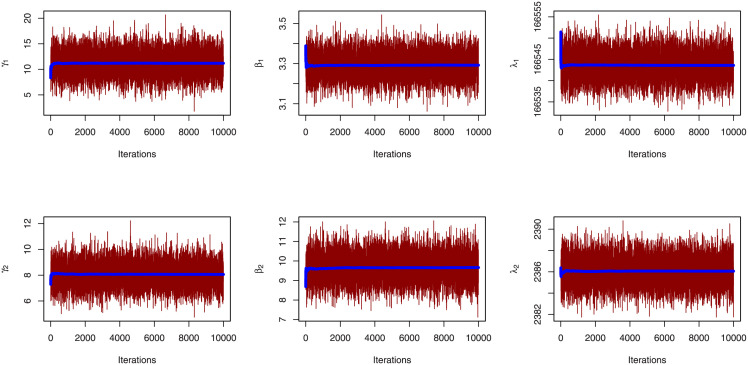
Trace plot of MCMC results with convergence lines.

**Fig 11:**
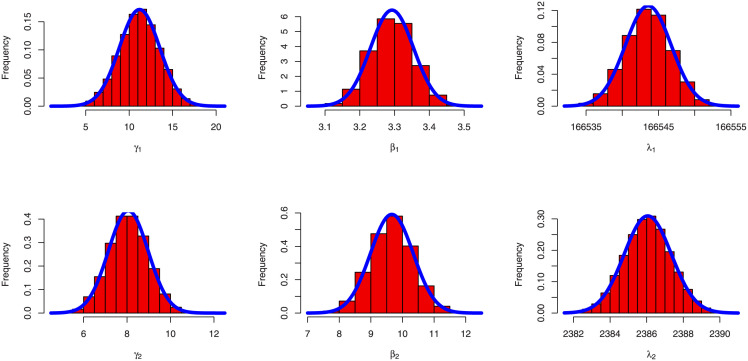
Histogram plot with density posterior curve of MCMC results.

**Fig 12:**
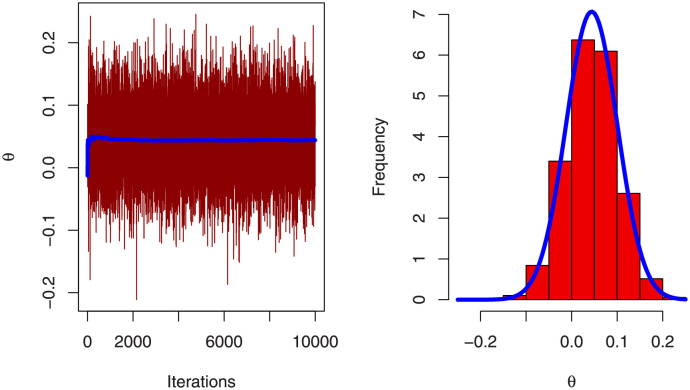
Trace with convergence line and histogram plot with density posterior curve of MCMC result for copula parameter.

MCMC summarized results for parameters of BFGMPLx model has been obtained in Table 8. Fig 10 displays the trace plot with convergence line for the BFGMPLx distribution’s parameters without copula parameter. Fig 11 demonstrates the symmetric normal distribution of the posterior density histogram for MCMC findings. Fig 12 trace plot with convergence line and posterior density histogram for MCMC of copula parameter *θ*.

**Table 8:** MCMC summarized results for parameters of BFGMPLx model.

	Min.	1st Qu.	Median	Mean	3rd Qu.	Max.	SE
*γ*_1_	1.7821	9.6366	11.2109	11.1903	12.7415	20.6666	2.3223
*β*_1_	3.0620	3.2496	3.2929	3.2926	3.3345	3.5440	0.0619
λ_1_	166532.7759	166541.4571	166543.5963	166543.5866	166545.7163	166555.4706	3.1720
*γ*_2_	4.7405	7.4438	8.0510	8.0579	8.6782	12.2197	0.9158
*β*_2_	7.1202	9.2090	9.6697	9.6634	10.1154	12.0607	0.6744
λ_2_	2381.7347	2385.1852	2386.0566	2386.0588	2386.9172	2390.7916	1.2885
*θ*	-0.2112	0.0060	0.0449	0.0441	0.0823	0.2454	0.0565

## 10 Conclusions

The bivariate power Lomax model, a new and more flexible extension of the bivariate distribution based on the FGM copula, is introduced in this paper. The suggested distribution is a significant and novel contribution to the field of bivariate modeling. It provides a robust and accurate tool for modeling heavy-tailed or skewed data and offers an improved way to model the dependence structure between two variables compared to traditional methods. Its fundamental mathematical characteristics are studied. Its hazard rate might be J-HR, IHR, Reversed J-HR, or Bathtube. It is demonstrated that the suggested model fits the positive (negative) quadrant dependence property. The parameter estimators, using likelihood and Bayesian methods, are derived, with the general conclusion that Bayesian estimation is better than its counterpart. A Monte Carlo simulation study is additionally offered to assess the behavior of the estimators. Asymptotic confidence intervals of the likelihood estimation and highest posterior density of the Bayesian estimation are derived for the parameters of this model. Finally, the significance and adaptability of the BFGMPLx distribution are discussed by analyzing medical data related to the duration of diabetes and serum creatinine. Analytical evaluations revealed that our BFGMPLx model had a satisfactory match when compared to other distributions.

## Supporting information

S1 Appendix (PDF)

## References

[1] Lomax K. S. Business failures: Another example of the analysis of failure. Journal of the American Statistical Association, 1954, 49, 847–852. doi: 10.1080/01621459.1954.10501239

[2] Hassan A. S., Al-Ghamdi A. S. Optimum step stress accelerated life testing for Lomax distrbution. Journal of Applied Sciences Research, 2009, 5, 2153–2164.

[3] Harris C. M. The Pareto distribution as a queue service discipline. Operations Research, 1968, 16, 307–313. doi: 10.1287/opre.16.2.307

[4] Holland O., Golaup A., and Aghvami A. Traffic characteristics oof aggregated module downloads for mobile terminal reconfiguration, IEE Proceedings-communications, 2006, 153, 683–690. doi: 10.1049/ip-com:20045155

[5] Campbell G., and Ratnaparkhi M. V. An application of lomax distributions in receiver operating characteristic (roc) curve analysis. Communications in Statistics–Theory and Methods, 1993, 22, 1681–1697. doi: 10.1080/03610929308831110

[6] Rady E. H. A., Hassanein W. A., and Elhaddad T. A. The power Lomax distribution with an application to bladder cancer data, SpringerPlus, 2016, 5, 1–22. doi: 10.1186/s40064-016-3464-y 27818876 PMC5074989

[7] Lai C. D. Constructions of continuous bivariate distributions. Journal of the Indian Society for Probability and Statistics, 2004, 8, 21–43.

[8] Nelsen R. B. An introduction to copulas. Springer Science Business Media, 2006.

[9] Sklar A. Random variables, joint distributions, and copulas. Kybernetica, 1973, 9, 449–460.

[10] Joe H. Multivariate models and dependence concepts. London: Chapman & Hall/CRC, 1997.

[11] El-Sherpieny E. S. A., Muhammed H. Z., & Almetwally E. M. (2022). Data Analysis by Adaptive Progressive Hybrid Censored Under Bivariate Model. Annals of Data Science, 1–42. doi: 10.1007/s40745-022-00455-z

[12] El-Sherpieny E. S. A., Muhammed H. Z., & Almetwally E. M. (2022). Bivariate Chen Distribution Based on Copula Function: Properties and Application of Diabetic Nephropathy. Journal of Statistical Theory and Practice, 16(3), 54. doi: 10.1007/s42519-022-00275-7

[13] El-Sherpieny E. S. A., Muhammed H. Z., & Almetwally E. M. Accelerated Life Testing for Bivariate Distributions based on Progressive Censored Samples with Random Removal. J. Stat. Appl. Probab, 2022, 11(2), 203–223.

[14] Gumbel E. J. Bivariate exponential distributions. Journal of the American Statistical, 1960, 55, 698–707. doi: 10.1080/01621459.1960.10483368

[15] Frank M. On the simultaneous associativity of functions. Journal of mathematical analysis and applications, 1979, 72, 352–360.

[16] Genest C., and MacKay J. Copulas, Archimax Distributions and Generation of Random Variates. Journal of Multivariate Analysis, 1986, 21, 100–120.

[17] Durante F., and Sempi C. Copulas: an introduction. CRC press, 2017.

[18] Morgenstern D. Einfache beispiele zweidimensionaler verteilungen. Mitteilingsblatt fur Mathematische Statistik, 1956, 8, 234–235.

[19] Farlie D. J. G. The performance of some correlation coefficients for a general bivariate distribution. Biometrika, 1960, 47, 307–323. doi: 10.2307/2333302

[20] Embrechts P., McNeil A. J., and Straumann D. Correlation and dependence in risk management: properties and pitfalls. Risk Management & Insurance Review, 2002, 5, 131–160.

[21] Gupta A. K., and Wong C. F. On three and five parameter bivariate beta distributions. Metrika, 1985, 32, 85–91. doi: 10.1007/BF01897803

[22] Vaidyanathan V. S., and Sharon Varghese A. Morgenstern type bivariate Lindley distribution. Statistics, Optimization & Information Computing, 2016, 4, 132–146.

[23] Almetwally E. M., and Muhammed H. Z. On a bivariate Fréchet distribution. Journal Statistics Appications Probability, 2020, 9, 1–21.

[24] El-Sherpieny E. S. A., Almetwally E. M., & Muhammed H. Z. Bivariate weibull-g family based on copula function: properties, bayesian and non-bayesian estimation and applications. Statistics, Optimization & Information Computing, 2022, 10(3), 678–709. doi: 10.19139/soic-2310-5070-1129

[25] Muhammed H. Z., El-Sherpieny E. S. A., and Almetwally E. M. Dependency measures for new bivariate models based on copula function. Information Sciences Letters, 2021, 10, 511–526. 10.18576/isl/100316

[26] Abulebda M., Pathak A. K., Pandey A., and Tyagi S. On a Bivariate XGamma Distribution Derived from Copula. Statistica, 2022, 82, 15–40.

[27] Hassan M. K., and Chesneau C. Bivariate Generalized Half-Logistic Distribution: Properties and Its Application in Household Financial Affordability in KSA. Mathematical and Computational Applications, 2022, 27, 72. Justel A., Peña D., and Zamar R. A multivariate Kolmogorov-Smirnov test of goodness of fit. Statistics & probability letters, 1997, 35, 251-259. doi: 10.3390/mca27040072

[28] Conway, D. A. Farlie-Gumbel-Morgenstern Distributions. Wiley StatsRef: Statistics Reference Online. 2014.

[29] Sreelakshmi N. An introduction to copula-based bivariate reliability Concepts. Communications in Statistics-Theory and Methods, 2018, 47, 996–1012. doi: 10.1080/03610926.2017.1316396

[30] Lehmann E. L. Some concepts of dependence. The Annals of Mathematical Statistics, 1966, 37, 1137–1153. doi: 10.1214/aoms/1177699260

[31] Basu A. P. Bivariate failure rate. Journal of American Statistical Association, 1971, 66, 103–104. doi: 10.1080/01621459.1971.10482228

[32] Johnson N. L., Kotz S. A vector multivariate hazard rate. Journal of Multivariate Analysis, 1975, 5, 53–66. doi: 10.1016/0047-259X(75)90055-X

[33] Shanbag D. N., Kotz S. Some new approaches to multivariate probability distributions. Journal of multivariate analysis, 1987, 22, 189–211. doi: 10.1016/0047-259X(87)90085-6

[34] Sankaran P. G., Nair U. On bivariate vitality functions. In Proceeding of National Symposium on Distribution Theory, 1991.

[35] Gupta R. D, and Kundu D. Generalized exponential distributions: statistical inferences. Journal Statistical Theory and Applications, 2002, 1, 101–118.

[36] Dey S., Singh S., Tripathi Y. M. and Asgharzadeh A. Estimation and prediction for a progressively censored generalized inverted exponential distribution. Statistical Methodology, 2016, 32, 185–202. doi: 10.1016/j.stamet.2016.05.007

[37] Hamdy A., & Almetwally E. M. Bayesian and Non-Bayesian Inference for The Generalized Power Akshaya Distribution with Application in Medical. Computational Journal of Mathematical and Statistical Sciences, 2023, 2, 31–51. doi: 10.21608/cjmss.2023.185497.1001

[38] Robert C. P., Casella G. The metropolis—Hastings algorithm. In Monte Carlo statistical methods (pp. 231–283). Springer, New York, NY, 1999.

[39] Chen M. H., and Shao Q. M. Monte Carlo estimation of Bayesian credible and HPD intervals. Journal of Computational and Graphical Statistics, 1999, 8, 69–92. doi: 10.2307/1390921

[40] Zhao J., Faqiri H., Ahmad Z., Emam W., Yusuf M., and Sharawy A. M. The lomax-claim model: bivariate extension and applications to financial data. Complexity, 2021, 2021, 1–17.

[41] Grover G., Sabharwal A., Mittal J. Application of Multivariate and Bivariate Normal Distributions to Estimate Duration of Diabetes. International Journal of Statistics and Applications, 2014, 4, 46–57.

