# Supplementary material for: Bivariate power Lomax distribution with medical applications
Source: PLoS One. 2023 Mar 8;18(3):e0282581. doi: 10.1371/journal.pone.0282581 (PMC9994725; doi:10.1371/journal.pone.0282581)
Supplement: S1 Appendix — (PDF) [file pone.0282581.s001.pdf]

11 Appendix

Proof of moment generatig function

$$M_{(x_1,x_2)}(t_1,t_2) = E\left(e^{t_1x_1}e^{t_2x_2}\right) = \int_0^\infty \int_0^\infty e^{t_1x_1}e^{t_2x_2} f(x_1,x_2)dx_1dx_2$$

By using the power series expression of e^{x_j} and $j=1,2$, we get

$$\begin{aligned}
M_{(x_1,x_2)}(t_1,t_2) = &\int_0^\infty \int_0^\infty \sum_{n_1=0}^\infty \frac{(t_1x_1)^{n_1}}{n_1!} \sum_{n_2=0}^\infty \frac{(t_2x_2)^{n_2}}{n_2!} f(x_1,x_2)dx_1dx_2\\
= &\sum_{n_1=0}^\infty \frac{t_1^{n_1}}{n_1!}\int_0^\infty x_1^{n_1} f(x_1)dx_1 \sum_{n_2=0}^\infty \frac{t_2^{n_2}}{n_2!}\int_0^\infty x_2^{n_2} f(x_2)dx_2\\
&+\theta \sum_{n_1=0}^\infty \frac{t_1^{n_1}}{n_1!}\int_0^\infty x_1^{n_1} f(x_1)dx_1 \sum_{n_2=0}^\infty \frac{t_2^{n_2}}{n_2!}\int_0^\infty x_2^{n_2} f(x_2)dx_2\\
&-2\theta \sum_{n_1=0}^\infty \frac{t_1^{n_1}}{n_1!}\int_0^\infty x_1^{n_1} f(x_1)dx_1 \sum_{n_2=0}^\infty \frac{t_2^{n_2}}{n_2!}\int_0^\infty x_2^{n_2} F(x_2)f(x_2)dx_2\\
&-2\theta \sum_{n_1=0}^\infty \frac{t_1^{n_1}}{n_1!}\int_0^\infty x_1^{n_1} F(x_1)f(x_1)dx_1 \sum_{n_2=0}^\infty \frac{t_2^{n_2}}{n_2!}\int_0^\infty x_2^{n_2} f(x_2)dx_2\\
&+4\theta \sum_{n_1=0}^\infty \frac{t_1^{n_1}}{n_1!}\int_0^\infty x_1^{n_1} F(x_1)f(x_1)dx_1 \sum_{n_2=0}^\infty \frac{t_2^{n_2}}{n_2!}\int_0^\infty x_2^{n_2} F(x_2)f(x_2)dx_2,
\end{aligned}$$

where

$$\begin{aligned}
\sum_{n_j=0}^\infty \frac{t_j^{n_j}}{n_j!}\int_0^\infty x_j^{n_j} f(x_j)dx_j &= \sum_{n_j=0}^\infty \frac{t_j^{n_j}}{n_j!}\int_0^\infty \gamma_j\beta_j\lambda_j^{\gamma_j} x_j^{n_j+\beta_j-1}(\lambda_j+x_j^{\beta_j})^{-\gamma_j-1}\\
&= \sum_{n_j=0}^\infty \frac{t_j^{n_j}}{n_j!}\beta_j\lambda_j^{\frac{n_j}{\beta_j}}\mathcal{B}(\frac{n_j}{\beta_j},\gamma_j-\frac{n_j}{\beta_j}),
\end{aligned}$$

and

$$\begin{aligned}
&\sum_{n_j=0}^\infty \frac{t_j^{n_j}}{n_j!}\int_0^\infty x_j^{n_j} F(x_j)f(x_j)dx_j =\\
&\sum_{n_j=0}^\infty \frac{t_j^{n_j}}{n_j!}\beta_j\lambda_j^{\frac{n_j}{\beta_j}}\mathcal{B}(\frac{n_j}{\beta_j},\gamma_j-\frac{n_j}{\beta_j}) - \frac{1}{2}\sum_{n_j=0}^\infty \frac{t_j^{n_j}}{n_j!}\beta_j\lambda_j^{\frac{n_j}{\beta_j}}\mathcal{B}(\frac{n_j}{\beta_j},2\gamma_j-\frac{n_j}{\beta_j}). \qquad (45)
\end{aligned}$$

Then

$$M_{x_1,x_2}(t_1,t_2) = \sum_{n_1=0}^{\infty} \frac{(t_1)^{n_1}}{n_1!} \frac{n_1}{\beta_1} \lambda_1^{\frac{n_1}{\beta_1}} \mathcal{B}(\frac{n_1}{\beta_1}, \gamma_1 - \frac{n_1}{\beta_1}) \sum_{n_2=0}^{\infty} \frac{(t_2)^{n_2}}{n_2!} \frac{n_2}{\beta_2} \lambda_2^{\frac{n_2}{\beta_2}}$$
$$\mathcal{B}(\frac{n_2}{\beta_2}, \gamma_2 - \frac{n_2}{\beta_2}) \left[1 + \theta - 2\theta\Omega_2 - 2\theta\Omega_1 + 4\theta\Omega_1\Omega_2\right],$$

To prove the product moments start with

$$\mu'_{r_1 r_2} = E\left(x_1^{r_1} x_2^{r_2}\right) = \int_0^{\infty} \int_0^{\infty} x_1^{r_1} x_2^{r_2} f(x_1,x_2) dx_1 dx_2,$$

$$\begin{aligned}
\mu'_{r_1 r_2} = & \int_0^{\infty} x_1^{n_1} f(x_1) dx_1 \int_0^{\infty} x_2^{n_2} f(x_2) dx_2 \\
& + \theta \int_0^{\infty} x_1^{n_1} f(x_1) dx_1 \int_0^{\infty} x_2^{n_2} f(x_2) dx_2 \\
& - 2\theta \int_0^{\infty} x_1^{n_1} f(x_1) dx_1 \int_0^{\infty} x_2^{n_2} F(x_2) f(x_2) dx_2 \\
& - 2\theta \int_0^{\infty} x_1^{n_1} F(x_1) f(x_1) dx_1 \int_0^{\infty} x_2^{n_2} f(x_2) dx_2 \\
& + 4\theta \int_0^{\infty} x_1^{n_1} F(x_1) f(x_1) dx_1 \int_0^{\infty} x_2^{n_2} F(x_2) f(x_2) dx_2,
\end{aligned}$$

where

$$\int_0^{\infty} x_j^{r_j} f(x_j) dx_j = \int_0^{\infty} \gamma_j \beta_j \lambda_j^{\gamma_j} x_j^{r_j + \beta_j - 1} (\lambda_j + x_j^{\beta_j})^{-\gamma_j - 1} = \beta_j \lambda_j^{\frac{r_j}{\beta_j}} \mathcal{B}(\frac{r_j}{\beta_j}, \gamma_1 - \frac{r_j}{\beta_j}),$$

and

$$\int_0^{\infty} x_j^{r_j} F(x_j) f(x_j) dx_j = \beta_j \lambda_j^{\frac{r_j}{\beta_j}} \mathcal{B}(\frac{r_j}{\beta_j}, \gamma_j - \frac{r_j}{\beta_j}) - \frac{1}{2} \beta_j \lambda_j^{\frac{r_j}{\beta_j}} \mathcal{B}(\frac{r_j}{\beta_j}, 2\gamma_j - \frac{r_j}{\beta_j}).$$

Then

$$\mu'_{r_1 r_2} = \frac{r_1}{\beta_1} \lambda_1^{\frac{r_1}{\beta_1}} \mathcal{B}(\frac{r_1}{\beta_1}, \gamma_1 - \frac{r_1}{\beta_1}) \frac{r_2}{\beta_2} \lambda_2^{\frac{r_2}{\beta_2}} \mathcal{B}(\frac{r_2}{\beta_2}, \gamma_2 - \frac{r_2}{\beta_2}) \left[1 + \theta - 2\theta\Upsilon_2 - 2\theta\Upsilon_1 + 4\theta\Upsilon_1\Upsilon_2\right].$$
